# The Coordinated Action of MYB Activators and Repressors Controls Proanthocyanidin and Anthocyanin Biosynthesis in *Vaccinium*

**DOI:** 10.3389/fpls.2022.910155

**Published:** 2022-06-24

**Authors:** Declan J. Lafferty, Richard V. Espley, Cecilia H. Deng, Andrew P. Dare, Catrin S. Günther, Laura Jaakola, Katja Karppinen, Murray R. Boase, Lei Wang, Henry Luo, Andrew C. Allan, Nick W. Albert

**Affiliations:** ^1^The New Zealand Institute for Plant and Food Research Limited, Palmerston North, New Zealand; ^2^School of Biological Sciences, The University of Auckland, Auckland, New Zealand; ^3^The New Zealand Institute for Plant and Food Research Limited, Auckland, New Zealand; ^4^Department of Arctic and Marine Biology, UiT The Arctic University of Norway, Tromsø, Norway; ^5^Norwegian Institute of Bioeconomy Research (NIBIO), Tromsø, Norway

**Keywords:** anthocyanin, proanthocyanidin, MYB, transcription factor, flavonoid, berry, repressor

## Abstract

*Vaccinium* berries are regarded as “superfoods” owing to their high concentrations of anthocyanins, flavonoid metabolites that provide pigmentation and positively affect human health. Anthocyanin localization differs between the fruit of cultivated highbush blueberry (*V. corymbosum*) and wild bilberry (*V. myrtillus*), with the latter having deep red flesh coloration. Analysis of comparative transcriptomics across a developmental series of blueberry and bilberry fruit skin and flesh identified candidate anthocyanin regulators responsible for this distinction. This included multiple activator and repressor transcription factors (TFs) that correlated strongly with anthocyanin production and had minimal expression in blueberry (non-pigmented) flesh. R2R3 MYB TFs appeared key to the presence and absence of anthocyanin-based pigmentation; *MYBA1* and *MYBPA1.1* co-activated the pathway while *MYBC2.1* repressed it. Transient overexpression of *MYBA1* in *Nicotiana benthamiana* strongly induced anthocyanins, but this was substantially reduced when co-infiltrated with *MYBC2.1*. Co-infiltration of *MYBC2.1* with *MYBA1* also reduced activation of *DFR* and *UFGT*, key anthocyanin biosynthesis genes, in promoter activation studies. We demonstrated that these TFs operate within a regulatory hierarchy where *MYBA1* activated the promoters of *MYBC2.1* and *bHLH2.* Stable overexpression of *VcMYBA1* in blueberry elevated anthocyanin content in transgenic plants, indicating that *MYBA1* is sufficient to upregulate the TF module and activate the pathway. Our findings identify TF activators and repressors that are hierarchically regulated by SG6 *MYBA1,* and fine-tune anthocyanin production in *Vaccinium.* The lack of this TF module in blueberry flesh results in an absence of anthocyanins.

## Introduction

Anthocyanins are plant-specific compounds, which are usually responsible for the blue, purple and red colors found in plants. They are some of the most abundant secondary metabolites in *Vaccinium* fruit and contribute to their health promoting properties (Khoo et al., 2017), leading to *Vaccinium* berries being described as “superfoods”. Blueberries (*Vaccinium* spp., mainly *Vaccinium corymbosum* and *Vaccinium virgatum*) are one of the most well-known and commercially cultivated *Vaccinium* species, while there are numerous species such as bilberry (*Vaccinium myrtillus*), which are harvested from the wild. The anthocyanin localization in the fruit of these species differs, with blueberries having a pale, colorless flesh while bilberries have a red, anthocyanin-rich flesh (Lafferty et al., 2022).

In *Vaccinium*, proanthocyanidins (PAs) are the most abundant secondary metabolite during early fruit development (Zifkin et al., 2012; Karppinen et al., 2016). Proanthocyanidins confer the astringent and bitter taste associated with unripe fruit, which deters consumption before seed maturity, while anthocyanin accumulation signals fruit ripening (Rauf et al., 2019). Both PAs and anthocyanins are produced *via* the flavonoid pathway, sharing many common biosynthetic steps. This includes the conversion of leucoanthocyanidin into anthocyanidin by a 2-oxoglutarate dependent dioxygenase known as anthocyanidin synthase (ANS) or leucoanthocyanidin dioxygenase (LDOX). While some species have a single *ANS/LDOX* gene, others have multiple gene family members that can be phylogenetically separated into distinct clades, which are referred to as *ANS* (anthocyanins) or *LDOX* (proanthocyanidins; Jun et al., 2018; Wang et al., 2021). The PA specific biosynthetic steps occur when anthocyanin reductase (ANR) converts anthocyanidins to epicatechin or when leucoanthocyanidin reductase (LAR) converts leucoanthocyanidins to catechin. As *Vaccinium* fruit ripen the PA concentrations decrease and anthocyanins begin to accumulate, seen as pigmentation development during ripening (Zifkin et al., 2012; Karppinen et al., 2016). The comitted step for anthocyanin biosynthesis involves the glycosylation of anthocyanidins by uridine diphosphate (UDP)-glucose: flavonoid-*O*-glycosyltransferase (UFGT).

The flavonoid pathway is regulated at the transcriptional level, with an MBW complex promoting transcription of biosynthetic genes, consisting of R2R3 MYB, bHLH and WDR proteins (Baudry et al., 2004; Albert and Allan, 2021). The MYB transcription factor (TF) determines the specificity of the complex for the different biosynthetic gene promoters. Subgroup (SG) 5 MYBs typically regulate PA biosynthesis, and have been characterized in a number of fruiting species, including blueberry, bilberry, grape and strawberry (Terrier et al., 2009; Schaart et al., 2013; Karppinen et al., 2021; Lafferty et al., 2022). These MYBs drive MBW activation of the PA specific *ANR* and *LAR* genes. SG6 MYBs are involved in anthocyanin regulation, upregulating flavonoid biosynthetic genes and the anthocyanin specific *UFGT*. These are key regulators for controlling anthocyanin production in fruit (Albert and Allan, 2021), including blueberry and bilberry (Plunkett et al., 2018; Karppinen et al., 2021). An additional class of MYBs, MYBPA1-type, are dual regulators of PA and anthocyanin biosynthesis in fruit and are required for full activation of both pathways (Karppinen et al., 2021; Lafferty et al., 2022). These activate genes common to PA and anthocyanin biosynthesis, but are not sufficient to activate these pathways, and require the SG5 and SG6 MYBs to activate the additional pathway-specific genes for metabolite production. Arabidopsis lacks a *MYBPA1* gene, but these MYBs appear to be important for species with more complex flavonoid profiles, such as are commonly found in fruit.

MYB repressors also contribute to PA and anthocyanin regulation. R3 MYB and SG4 R2R3 MYBs are two well-characterized classes of MYB repressors with distinct functions. R3 MYBs are small proteins that lack activation and repression motifs, yet have retained their bHLH binding domain (Zimmermann et al., 2004). When R3 MYBs bind bHLH proteins, this limits the formation of activator MBW complexes, referred to as passive repression (Zhang et al., 2009; Albert et al., 2014). While these repressors are best characterized for their roles that affect flower color and patterning (petunia, *Mimulus*; Albert et al., 2014; Ding et al., 2020), R3 MYBs have also been characterized and shown to inhibit anthocyanin production in fruiting species such as tomato (Colanero et al., 2018). Two R3 MYBs have been identified in bilberry and bog bilberry (*V. uligonosum*), but have yet to be functionally characterized (Primetta et al., 2015; Zorenc et al., 2017). The SG4 R2R3 MYBs function through active repression, as they have an ethylene-responsive element binding factor (ERF) associated amphiphilic repression (EAR) motif (Albert et al., 2014). This is recognized by TOPLESS (TPL), which recruits chromatin remodeling factors to the promoter region and inhibits transcription at the epigenetic level (Kagale and Rozwadowski, 2011). SG4 MYBs often have TLLLFR motifs that contribute to repressive activity (Matsui et al., 2008). Inhibition of the production of both PAs and anthocyanins by SG4 MYB repressors has been reported in a number of fruiting species, including apple, grape, peach and strawberry (Aharoni et al., 2001; Gao et al., 2011; Pérez-Díaz et al., 2016; Zhou et al., 2019; Zhu et al., 2019).

Hierarchical regulation, where a TF activates the expression of additional TFs, is important for flavonoid regulation in some species, including fruit systems. This has been observed for MYB repressors, where activator MYBs promoted their expression as part of a feedback repression loop, controlling the extent of activation for each pathway (Albert et al., 2014). This has also been reported for SG4 MYBs in petunia, poplar, *Medicago truncatula* and citrus (Albert et al., 2014; Jun et al., 2015; Yoshida et al., 2015; Huang et al., 2019) and for R3 MYBs in petunia and tomato (Albert et al., 2014; Yan et al., 2020). Hierarchy between activator TFs also occurs, with activator MYBs upregulating bHLH genes in Arabidopsis, kiwifruit, clover, Antirrhinum, tobacco and petunia (Baudry et al., 2006; Xu et al., 2013; Albert et al., 2014, 2021; Albert, 2015; Montefiori et al., 2015; Liu et al., 2021). Recently, the hierarchical regulation of blueberry *MYBPA1.1* by both SG5 and SG6 MYB activators was reported (Lafferty et al., 2022). This was the first report of a SG6 MYB regulating MYBPA1-type MYBs, while their regulation by SG5 MYBs had been seen in apple, grape and poplar (Terrier et al., 2009; James et al., 2017; Wang et al., 2018). It is not currently known how this aspect of flavonoid regulation fits within the wider module of anthocyanin regulators, or whether any hierarchical regulation between members exists. Furthermore, flavonoid repressors have not yet been functionally characterized in *Vaccinium* spp.

The flavonoid profile of *Vaccinium* fruit varies during both fruit development and ripening. We hypothesize that coordinated expression of multiple TFs is required for full activation of the PA and anthocyanin pathways. Knowledge of these processes may help resolve the causal regulatory changes in bilberry that confer red flesh. To study this, we performed differential expression analysis on an RNA-sequencing (RNA-seq) dataset to identify candidate anthocyanin regulators. This dataset included both a blueberry and bilberry fruit developmental series, with skin and flesh tissues separated. Candidate genes were functionally characterized and hierarchical regulation was investigated. Finally, stable overexpression of the SG6 *VcMYBA1* in blueberry was carried out to determine whether expression of this TF was sufficient for full activation of the anthocyanin pathway *via* activation of secondary transcriptional regulators.

## Materials and Methods

### Plant Material and Sampling

Northern Highbush blueberry (*V. corymbosum*) ‘Nui’ fruit and wild bilberry fruit were obtained as described previously, representing a developmental series from stage 4 to 8 (Günther et al., 2020; Lafferty et al., 2022). Additionally, stage 1–8 whole berry samples from “Nui” blueberry were sampled for qRT-PCR (Lafferty et al., 2022).

### RNA-Sequencing, Differential Expression and Correlation Analysis

The raw RNAseq datasets used for this study were described previously for blueberry (Günther et al., 2020) and bilberry (Wu et al., 2021) and were deposited on NCBI under PRJNA591663 and PRJNA739815, respectively. The analysis of raw sequence datasets was performed and mapped to a *V. corymbosum* “Draper” genome (Colle et al., 2019). Raw read counts were normalized *via* the median of ratios method (Love et al., 2014).

Differential gene expression analysis was performed with the “*DESeq2”* R package (Love et al., 2014). Transcripts with an adjusted *P*-value (padj) less than 0.01 and log2-fold change greater than 2 were determined to be highly differentially expressed genes (HDEG). Multiple DESeq2 comparisons were performed and overlaid with each other using the “*VennDiagram*” R package (Chen and Boutros, 2011). HDEGs can be found in Supplementary Table S1.

Highly differentially expressed genes were functionally annotated with Mapman bin numbers using Mercator4 V2.0, and flavonoid biosynthetic genes were visualized using the MapMan version 3.5.1R2 software (Schwacke et al., 2019), displaying the log-fold change between S7 blueberry and bilberry flesh. A table displaying the HDEGs annotated as TFs was produced in R using the “*data.table*” and “*formattable*” R packages. The closest Arabidopsis homolog was obtained by a BLAST of the gene ID against the Araport11 protein sequences dataset using TAIR BLAST 2.9.0 +.

Heatmaps were constructed using the “gplots” R package (Warnes et al., 2009). The blueberry genome includes all four haplotypes, and therefore the same gene may be represented by four gene IDs in the analysis. When this occurred, the gene ID with the greatest count data was chosen. Expression was displayed as the *Z*-score, which represents the number of standard deviations below (negative score) or above (positive score) the data mean for the given gene in each tissue, across development. The blueberry genome gene IDs for flavonoid biosynthetic and regulatory genes analyzed in this study are provided in Supplementary Table S2.

Correlation analysis was performed for each tissue type by calculating Spearman rank correlations on normalized read counts using R version 4.0.5 and the *“Hmisc*” package (Harrell and Dupont, 2017). The output was visualized using the “*corrplot*” package (Wei and Simko, 2017).

### Cloning of TFs and Promoters

Cloning of *GUS, PpbHLH3, VcMYBA1, VcMYBPA1.1* and *VcMYBPA2.2* is described previously (Lafferty et al., 2022). Promoter cloning of *V. virgatum DFR* and *UFGT* and *V. corymbosum ANR*, into the pGreenII 0800-LUC vector was described previously (Lafferty et al., 2022). The *VcMYBR3.1* sequence was identified in the blueberry reference transcriptome (*V. corymbosum* RefTrans V1), from the Genome Database for *Vaccinium*, based on its similarity to *VuMYBR3* (KT186105.1). The *VcMYBC2.1* gene ID was identified from the blueberry genome based on the differential expression analysis (Supplementary Table S5). Overexpression constructs for *VcMYBR3.1* and *VcMYBC2.1* and promoter constructs for *VcMYBR3.1pro, VcMYBC2.1pro* and *VcbHLH2pro* were made as described in Lafferty et al. (2022) and gene-specific primers used are provided in Supplementary Table S3.

### Transient Transformation of Tobacco for Anthocyanin and Proanthocyanidin Production

*Agrobacterium* strains containing effector (*35S:TF*) constructs were prepared for infiltration as previously described (Lafferty et al., 2022). *Agrobacterium* mixtures, containing 1/3 volume of up to three *Agrobacterium* strains were prepared. This contained the activator MYB TF in combination with *PpbHLH3* (Zhou et al., 2018) and either the repressor MYB TF or *35S:GUS* (negative control). Approximately 500 μl of the mixture was infiltrated into the abaxial surface of young *Nicotiana benthamiana* ‘Northern Territory’ leaves using a 1 ml needleless syringe. For each treatment three biological replicates were performed, consisting of an infiltrated leaf on separate plants (Supplementary Figure S5). *N. benthamiana* growing conditions and *Agrobacterium* infiltration were as described previously (Lafferty et al., 2022). Leaves were photographed 5 days post-infiltration.

### Promoter Activation Assays

For promoter activation assays, *Agrobacterium* cultures were prepared with 1/10 volume of promoter construct and 3/10 volume of up to three effector constructs, harboring effector TFs or the GUS negative controls. Mixtures were infiltrated into young *N. benthamiana* (“LAB” strain) leaves. Three leaves were infiltrated per treatment, as biological replicates. Dual luciferase assays were performed with DLAR-2B reagents (Targeting Systems), following the manufacturer’s instructions, using a Tecan Spark^®^ 20 M multimode microplate reader. Values and error bars represent the mean of three biological replicates and ± standard error, respectively. Letters indicate significant differences assessed by one-way ANOVA and *post hoc* Tukey’s LSD tests (*P* < 0.05) performed on log-transformed data.

### Stable Transformation of Blueberry

The *35S_pro_:MYBA1:OCS* cassette from pKES8 (Plunkett et al., 2018) was cloned into pART27 binary vector (pKES10) and transformed into *Agrobacterium tumefaciens* GV3101 by electroporation. Agrobacteria were grown overnight in lysogeny broth to A_600_ = 0.6–0.8, harvested by centrifugation and resuspended in AB *vir* gene pre-induction medium (Gelvin and Liu, 1994) containing 200 μm acetosyringone to A_600_ = 0.2. Agrobacterium suspensions were then cultured for 4 h at 28°C and 250 rpm in an orbital shaker.

*In vitro* stock cultures of *V. corymbosum* “Draper” × “Legacy” were established from shoot tip and single-node cuttings of glasshouse plants were grown on “micropropagation” medium. Media compositions are provided in Supplementary Table S6. Leaf blade explants were cut transversely several times to produce more sites for callus and shoot regeneration, and pre-cultured for 2 days on ‘co-cultivation’ medium. Pre-cultured leaf blade explants were inoculated with agrobacteria by swirling the AB culture in a 50 ml Falcon tube for 10 min in an orbital shaker. Explants were transferred to filter paper on “co-cultivation medium” and cultured at 22°C for 6 days in the dark. After co-cultivation, explants were washed three times by shaking with 40 ml of liquid “regeneration” medium (“selection” medium without antibiotics or agar) in a 50 ml Falcon tube, rinsed with liquid “regeneration” medium containing 500 mg L^−1^ cefotaxime, and transferred to solid ‘selection’ medium without filter paper, and kept in the dark for 2 weeks at 25°C. Explants were transferred to new ‘selection’ media every 3 weeks for 5–6 months. Red calli were visible on transformed explants and green shoots on regeneration controls by 4 months after the experiment was set up. As shoots regenerated on explants, they were labeled with explant origin and transferred to “shoot proliferation” medium. Roots were initiated by transferring shoots onto “rooting” medium.

### cDNA Synthesis and qRT-PCR Analysis for “Nui” Fruit Developmental Series and *35S:MYBA1* Blueberry Transformants

*Vaccinium corymbosum* “Nui” fruit from stages 1 to 3 were collected between November and December 2020 to supplement the existing fruit development series. Leaf and stem tissues of regenerating transgenic shoots were obtained from three independent transgenic lines and two regeneration controls, with three biological replicates. Total RNA was extracted from approximately 50 mg of frozen, ground leaf tissue, using the Spectrum™ Plant total RNA kit (Sigma-Aldrich, United States of America) with minor modification, as described previously (Günther et al., 2020). cDNA synthesis and qRT-PCR was performed with three biological replicates, normalized to the geometric mean of *GAPDH* and *Actin* (Lafferty et al., 2022). Primer efficiencies were verified using serial dilution with the gene-specific primers listed in Supplementary Table S4.

## Results

### Differential Expression Analysis of Blueberry and Bilberry Fruit Developmental Series Reveals Candidate Anthocyanin Regulators

A developmental series of berries was obtained as described previously (Lafferty et al., 2022). This consisted of blueberry (*V. corymbosum*) and bilberry (*V. myrtillus*) fruit, ranging from immature, small, green fruit (S4) to fully ripened blue fruit (S8). Anthocyanin pigmentation was restricted to the skin of blueberry and was present in both bilberry skin and flesh, developing at S5 and increasing as fruit ripened (Lafferty et al., 2022). The skin and flesh were separated and these samples were used for RNA sequencing (RNA-seq) analysis. RNA-seq reads were mapped against a tetraploid blueberry genome assembly, which included all four haplotypes (Colle et al., 2019).

**Figure 1:**
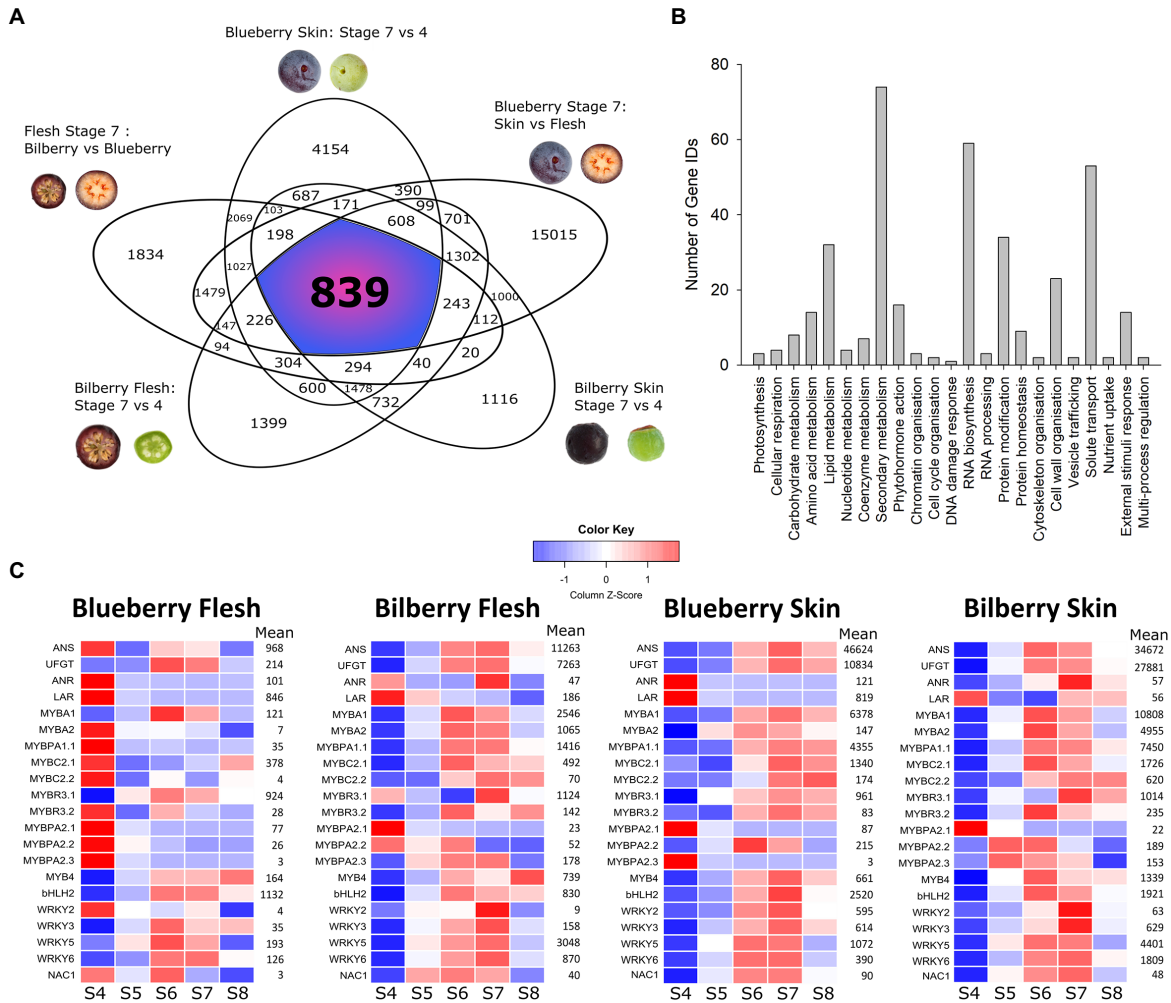
Differential expression analysis of the blueberry and bilberry RNA-seq dataset. **(A)** Highly differentially expressed genes (HDEGS) were identified by comparing high and low anthocyanin-producing tissues. HDEGs were described as having a log2-fold change greater than 2 in high-anthocyanin tissues, with an –adjusted *p*-value < 0.01. HDEGs consistently present across each comparison were identified and visualized as a Venn diagram. Comparisons were between blueberry skin S7 vs. S4, blueberry S7 skin vs. flesh, bilberry skin S7 vs. S4, bilberry flesh S7 vs. S4 and blueberry vs. bilberry S7 flesh. **(B)** HDEGs consistent across all comparisons were annotated using Mercator4 (V2.0), which assigned Mapman bin numbers. This was visualized as a bar graph in SigmaPlot (V14.0). **(C)** The developmental expression patterns of candidate anthocyanin transcription factors (TFs), SG5 MYBPA1-TFs and representative biosynthetic genes were visualized as a heatmap. *ANS* and *UFGT* represented anthocyanin biosynthesis while *ANR* and *LAR* represented proanthocyanidin (PA) biosynthesis. Raw counts were normalized *via* the median of ratios method. Count data were visualized as *Z*-scores, which calculated how many standard deviations a data point was from the mean of a gene across all samples in the given tissue, with blue and red representing negative and positive *Z*-scores, respectively. Mean represents the mean counts for each gene, in each tissue type, across development.

Highly differentially expressed genes (HDEGs) had a log_2_-fold change greater than 2, with an adjusted *P*-value < 0.01. Comparisons were performed between ripe (S7) and unripe (S4) blueberry skin and bilberry skin and flesh, ripe (S7) blueberry skin and flesh, and ripe (S7) blueberry and bilberry flesh (Figure 1A). These comparisons were structured to filter out genes that were involved in ripening- and tissue-specific processes. In total, 839 genes were identified as being HDEGs (Supplementary Table S1). Their deduced amino acid sequences were extracted from the blueberry genome and annotated using Mercator4 V2.0 (Schwacke et al., 2019). The majority of HDEGs with annotation were predicted to have roles in either secondary metabolism, RNA biosynthesis or solute transport (Figure 1B), and 403 HDEGs were unannotated.

The expression profiles of general flavonoid and anthocyanin biosynthetic genes and transporters were compared between blueberry and bilberry ripe (S7) flesh. The log_2_-fold change of these genes was visualized (Supplementary Figure S1). Phenylpropanoid and flavonoid biosynthetic and transporter genes were highly upregulated in bilberry S7 flesh, with *PAL, C4H, 4CL, CHS, F3′5′H, ANS, UFGT, GST,* and *MATE* having approximately five log_2_-fold higher expression.

A number of HDEGs were annotated as being TFs (Supplementary Table S5). These were further annotated by finding the best BLAST hit to Arabidopsis. Up to four gene IDs may correspond to the same gene owing to all four haplotypes being represented in the tetraploid blueberry genome. Taking this into consideration, eight unique MYB genes were present in the analysis. Phylogenetic analysis identified the subgroups to which these R2R3 MYBs or R3 MYBs belonged (Supplementary Figure S2). This included the previously characterised SG6 *VcMYBA1* and MYBPA1-type *VcMYBPA1.1* (Zifkin et al., 2012; Plunkett et al., 2018). An additional SG6 MYB, named *VcMYBA2*, was present, which contained the [R/K]P[R/Q][P/R]RTF SG6 motif (Plunkett et al., 2018). Two SG4 MYBs were identified, named *VcMYBC2.1* and *VcMYBC2.2*, and these contained both the [L/F]PDLN[L/F]x EAR and TLLLFR repression motifs (Ohta et al., 2001; Matsui et al., 2008). Additionally, there was a SG1 MYB, named *VcMYB4* and an R3 MYB, named *VcMYBR3.2.* Furthermore, there were four unique WRKY TFs, *VcWRKY2, VcWRKY3, VcWRKY5* and *VcWRKY6*, two bHLH TFs, and a NAC TF (Supplementary Table S5).

### Expression and Correlation Analysis of Biosynthetic Genes and Candidate Anthocyanin Regulators

The expression profiles of flavonoid biosynthetic genes and candidate TFs, found by the differential expression analysis, were visualized (Figure 1C; Supplementary Figure S3). An anthocyanin related bHLH (*bHLH2*) and the SG5 R2R3 MYB PA regulators (*MYBPA2.1–3*), identified previously, were included (Günther et al., 2020; Lafferty et al., 2022). Additionally an R3 MYB, *VcMYBR3.1*, was included based on its sequence similarity to other R3 MYBs with established roles in anthocyanin regulation from other plants (Zhu et al., 2009; Albert et al., 2014; Primetta et al., 2015). The general flavonoid biosynthetic genes *PAL, C4H, CHS, CHI, F3H, F3*′*H, F3*′*5*′*H, DFR, LDOX,* and *ANS*, transporters *GST* and *MATE8* and the anthocyanin specific *UFGT* all had similar expression profiles to each other (Figure 1C; Supplementary Figure S3). *ANS* and *LDOX* genes in blueberry were distinguished phylogenetically (Supplementary Figure S4). In blueberry skin, and bilberry skin and flesh, their transcript abundance increased at S6, peaked at S7 and then declined slightly at S8. This correlated with visible pigmentation (Lafferty et al., 2022), with this pattern described as being anthocyanin-related. In blueberry flesh, the expression of these genes was much lower, shown by the mean of the normalized count data. In blueberry flesh, transcript abundance for *PAL, C4H, CHS, CHI, F3H, F3′5′H, DFR, LDOX,* and *MATE* was highest in blueberry flesh at S4 (when PAs were probably still being synthesized), and declined as ripening proceeded. The PA specific biosynthetic genes *ANR* and *LAR* also shared this expression pattern. In both species, the mean count data were higher in the skin than the flesh and many genes, *CHS, DFR, UFGT,* and *GST* in particular, had higher count data in bilberry skin than in blueberry skin.

In blueberry skin and bilberry skin and flesh many candidate TFs also had an anthocyanin related expression pattern (Figure 1C). This included *MYBA2, MYBPA1.1, MYBC2.1, MYBC2.2, MYBR3.2, WRKY2,* and *NAC1*. Furthermore, *MYBA1, MYBR3.1, MYB4, WRKY3, WRKY5, WKRY6,* and *bHLH2* had an anthocyanin related expression pattern in all tissues, including blueberry flesh. These TFs had much lower mean counts (normalized *via* the median of ratios method) in blueberry flesh, with the exception of *MYBR3.1* and *bHLH2,* which had similar mean count data to bilberry flesh. The mean count data of these TFs were generally much higher in skin tissues, aligning with observations for the candidate genes. The SG5 *MYBPA2.1* was most highly expressed at S4 in all tissues, corresponding to PA biosynthesis, while *MYBPA2.3* showed this expression profile in blueberry skin and flesh. *MYBPA2.2* and *MYBPA2.3* had a more ripening-related expression pattern, but only in bilberry. The mean count data of the SG5 MYBs was relatively low in all tissues.

*MYBA1* and *MYBPA1.1* are key anthocyanin regulators in *Vaccinium* (Karppinen et al., 2021; Lafferty et al., 2022). Correlation analysis was performed to identify which candidate TFs strongly correlated with the expression of these TFs and of the anthocyanin related biosynthetic genes *ANS* and *UFGT* (Supplementary Figure S5). In blueberry skin and bilberry skin and flesh, *bHLH2* consistently correlated with these genes, forming an anthocyanin-related cluster. In both bilberry tissues, this cluster also included *MYBC2.1* and in bilberry skin *MYBA2, MYB4, MYBR3.2* and *WRKY5* were additionally present. In blueberry skin, the anthocyanin-related cluster contained *MYB4, MYBR3.1, WRKY2, WRKY3,* and *NAC1*. Blueberry flesh lacked many of the significant gene correlations seen in the other tissues, although significant correlations between *UFGT, MYBA1, MYBR3.1,* and *bHLH2* were present.

### SG4 and R3 MYB Repressors Inhibit Both the PA and Anthocyanin Biosynthesis Pathways

Two MYB repressors were analyzed further to elucidate their role in anthocyanin regulation. *MYBC2.1* was identified in the differential expression and phylogenetic analysis as a candidate SG4 MYB repressor of anthocyanin biosynthesis (Figure 1C; Supplementary Figure S2). An R3 MYB repressor, *MYBR3.1*, was chosen as it was expressed in blueberry fruit flesh, where it may contribute to the lack of anthocyanins in this tissue. *MYBR3.1* was not identified as an HDEG in the transcriptomic analysis due to its expression in blueberry flesh (Figure 1C). Although both *MYBC2.2* and *MYBR3.2* were identified as being HDEGs, they were not analyzed further due to their minimal expression in fruit tissues. Gene expression analysis, *via* qRT-PCR, across a full blueberry developmental series revealed that *VcMYBC2.1* and *VcMYBR3.1* were expressed highly during early development (S1–S3; Supplementary Figure S6). Because of this expression profile, it was hypothesized that these candidate TFs could also repress the PA pathway.

**Figure 2:**
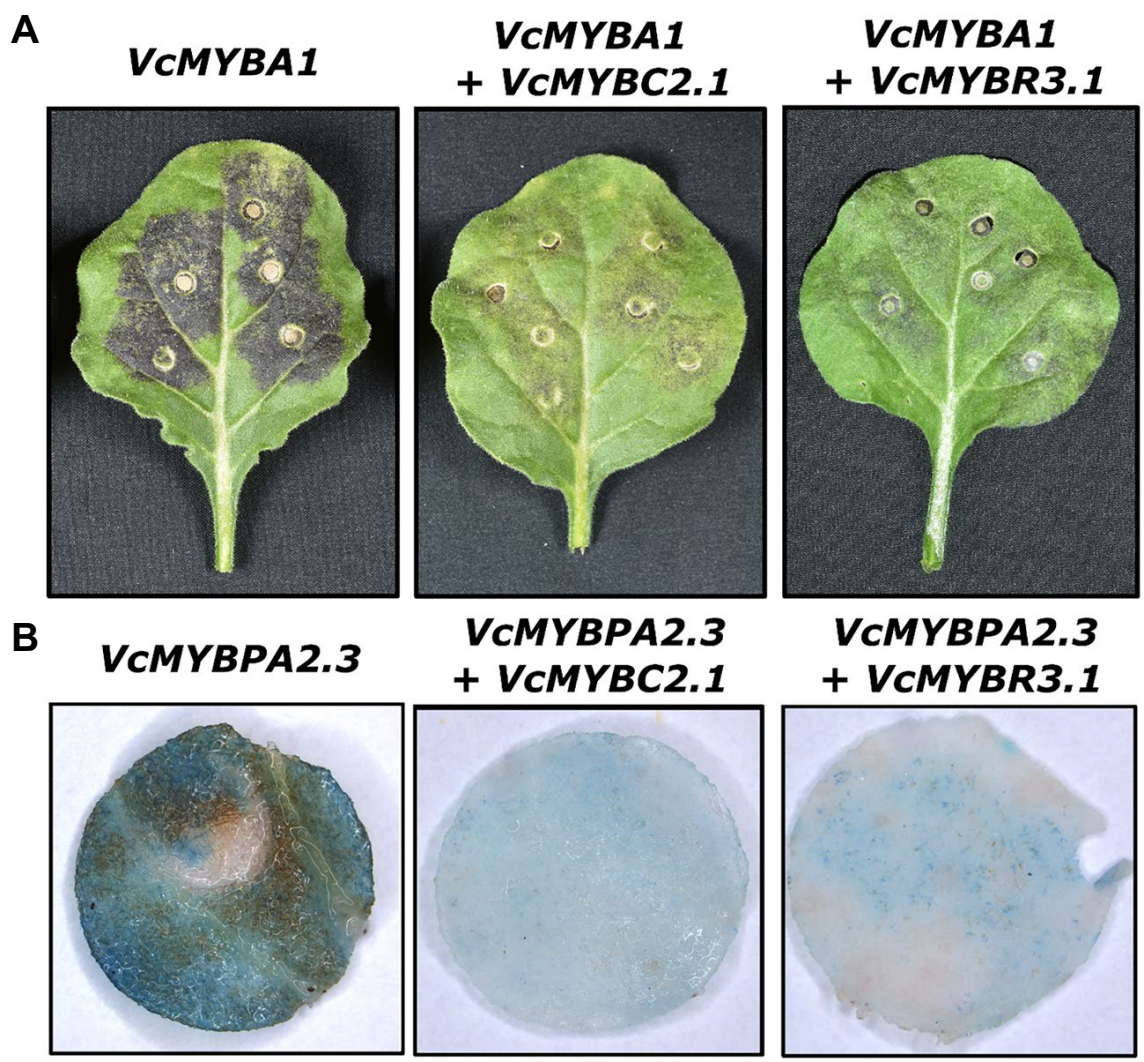
VcMYBC2.1 and VcMYBR3.1 inhibit anthocyanin and proanthocyanidin accumulation. Representative phenotypes of *Agrobacterium* infiltrated *Nicotiana benthamiana* leaves expressing MYB activators and *PpbHLH3*, and co-expressing the repressors *VcMYBC2.1* or *VcMYBR3.1*. The SG6 MYB activator *VcMYBA1* was used for anthocyanin accumulation assays **(A)** while the SG5 MYB activator *VcMYBPA2.3* was used for proanthocyanidin accumulation assays **(B)** Leaves were photographed 7 days post infiltration. For proanthocyanidin accumulation studies, 1 cm diameter leaf discs were taken and DMACA stained before being photographed.

*VcMYBC2.1* and *VcMYBR3.1* were isolated from blueberry and cloned into overexpression vectors for functional analyses. The MYB activators VcMYBA1 and VcMYBPA2.2 regulate anthocyanin and proanthocyanidin biosynthesis, respectively, and function within MBW complexes (Karppinen et al., 2021; Lafferty et al., 2022). We were unable to include *VcbHLH2* in these assays because despite amplifying *bHLH2* cDNAs from blueberry (and bilberry), we found this sequence was unstable in plasmids, possibly due to a microsatellite repeat within exon six. Therefore, we used the orthologue from *Prunus persica*, *PpbHLH3*. Transient overexpression of either *VcMYBA1* or *VcMYBPA2.2* in *N. benthamiana* leaves, with *PpbHLH3*, resulted in strong anthocyanin and PA accumulation, respectively (Figure 2; Supplementary Figure S7). The presence of PAs was observed using *p*-dimethylaminocinnamaldehyde (DMACA) staining. Co-infiltration of either *VcMYBC2.1* or *VcMYBR3.1* with these MYB activators substantially reduced the amount of pigmentation and PA produced.

**Figure 3:**
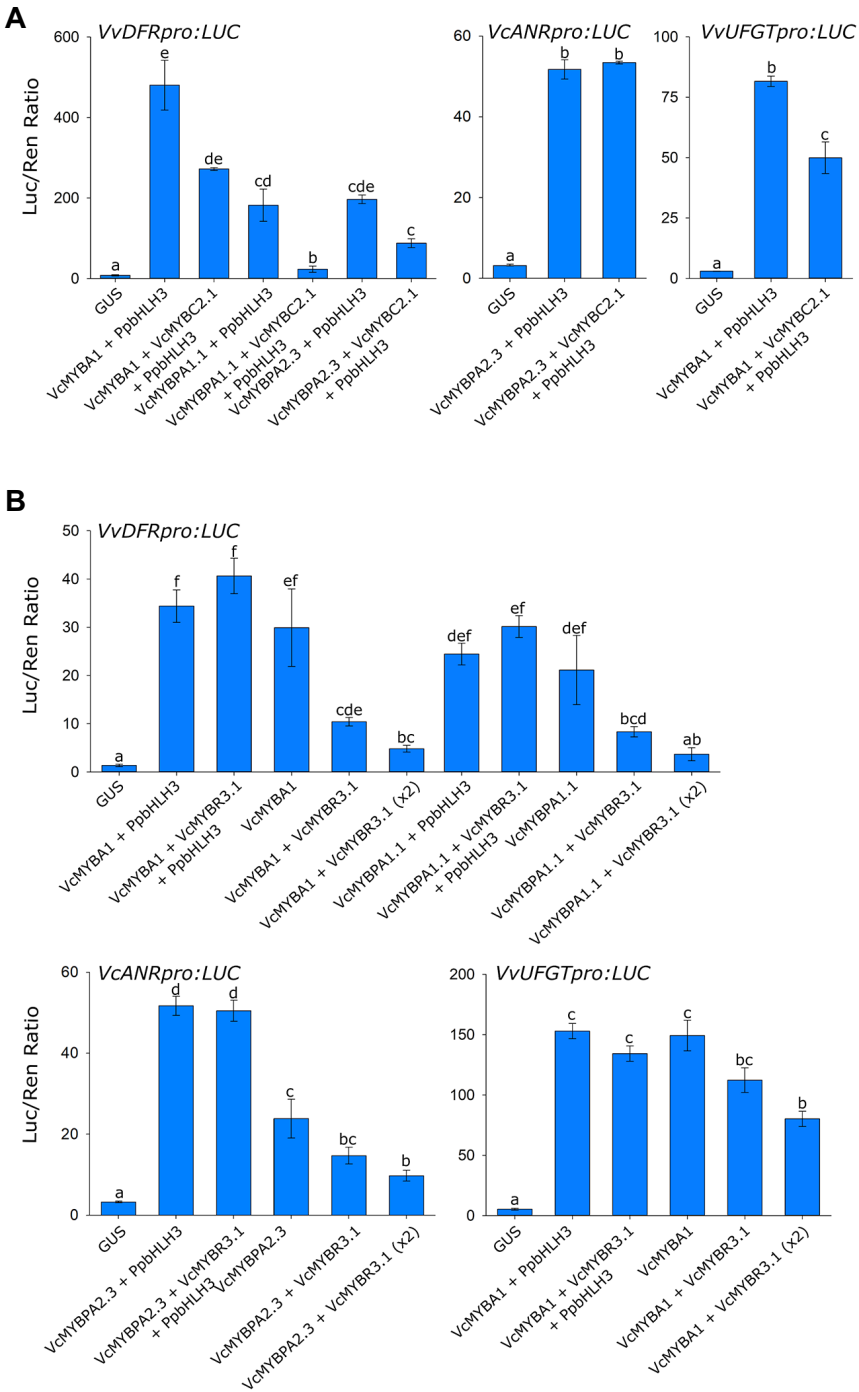
VcMYBC2.1 and VcMYBR3.1 repress anthocyanin and proanthocyanidin biosynthetic gene expression. Promoter activation assays were performed in *Nicotiana benthamiana* leaves *via Agrobacterium*-mediated co-infiltration of MYB activators and *PpbHLH3*, with and without either **(A)** *VcMYBC2.1* or **(B)** *VcMYBR3.1*, against the *DFR*, *ANR* and *UFGT* promoter:Luciferase constructs. *GUS* was used as a negative control. Values represent the mean of three biological replicates. Error bars show ± SEM. Letters indicate significant differences between treatments, assessed by one-way ANOVA and *post hoc* Tukey’s test (*P* < 0.05) on log-transformed data.

Promoter activation assays were performed on the *Vaccinium DFR, ANR* and *UFGT* promoters to assess the repression activity of these TFs (Figure 3). *VcMYBA1* and *VcMYBPA1.1* significantly activated *DFRpro*, *VcMYBPA2.2* significantly activated *ANRpro* and *VcMYBA1* significantly activated *UFGTpro,* either with and without *PpbHLH3*. The addition of *VcMYBC2.1* in transient infiltrations significantly reduced activation of the *DFRpro* by *VcMYBA1, VcMYBPA1.1* and *VcMYBPA2.3* (all with *PpbHLH3*). Furthermore, there was significantly lower activation of *UFGTpro* when *VcMYBC2.1* was co-infiltrated with *VcMYBA1* and *PpbHLH3*. Co-infiltration of *VcMYBPA2.3* and *PpbHLH3* activated the *ANRpro* and addition *VcMYBC2.1* had no effect on this activation. Combining *VcMYBR3.1* with activator MYBs and *PpbHLH3* had no effect on promoter activation. R3-MYB repressors are proposed to function by binding and titrating bHLH proteins (Albert et al., 2014), therefore the effects of altering the amount of supplied bHLH with *VcMYBR3.3* was examined. Removal of *PpbHLH3* from MYB activator and repressor co-infiltrations resulted in a significant drop in activation of all promoters, which was further reduced when the amount of *VcMYBR3.1* co-infiltrated was doubled.

### Proanthocyanidin and Anthocyanin TFs Are Hierarchically Regulated

**Figure 4:**
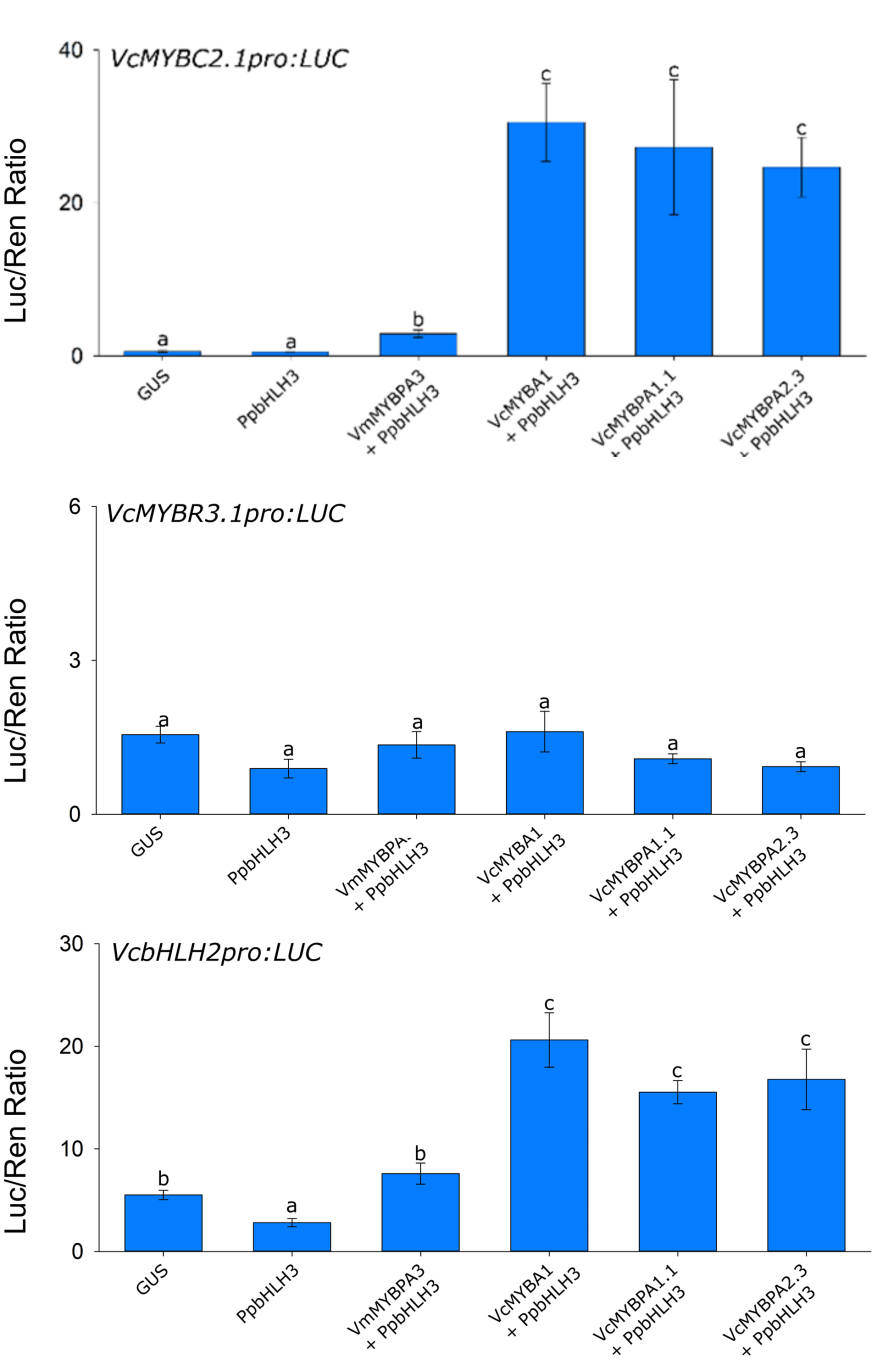
Hierarchical regulation of transcription factors by MYB activators. Promoter activation assays were performed in the leaves of *N. benthamiana via Agrobacterium-*mediated co-infiltration of SG5, SG6 and MYBPA1-type MYB activators, with *PpbHLH3*, against the *VcMYBC2.1, VcMYBR3.1, VcMYBPA1.1* and *VcbHLH2* promoter:Luciferase constructs. Values represent the mean of three biological replicates. Error bars are ± SEM. Letters indicate significant differences between treatments, assessed by one-way ANOVA and *post hoc* Tukey’s test (*P* < 0.05) on log-transformed data.

The correlation of *MYBC2.1, MYBR3.1,* and *bHLH2* with the *MYBA1* activator, in both blueberry and bilberry (Supplementary Figure S5), suggests they may be regulated by MYBA1 in an activator hierarchy. The three TFs are also involved in PA regulation and may also be regulated by MYBPA2.2 during early stages of fruit development. The promoters of the genes were isolated and cloned into dual luciferase vectors for promoter activation assays. All treatments included *PpbHLH3*, which was incapable of activating any promoters by itself. The *VcMYBPA2.2*, *VcMYBA1,* and *VcMYBPA1.1* activators significantly activated *VcMYBC2.1pro* and *VcbHLH2pro* (Figure 4). No TF combinations assayed against the *VcMYBR3.1pro* were able to induce activity of this promoter.

### *VcMYBA1* Overexpression in Blueberry Induces Anthocyanin Production

**Figure 5:**
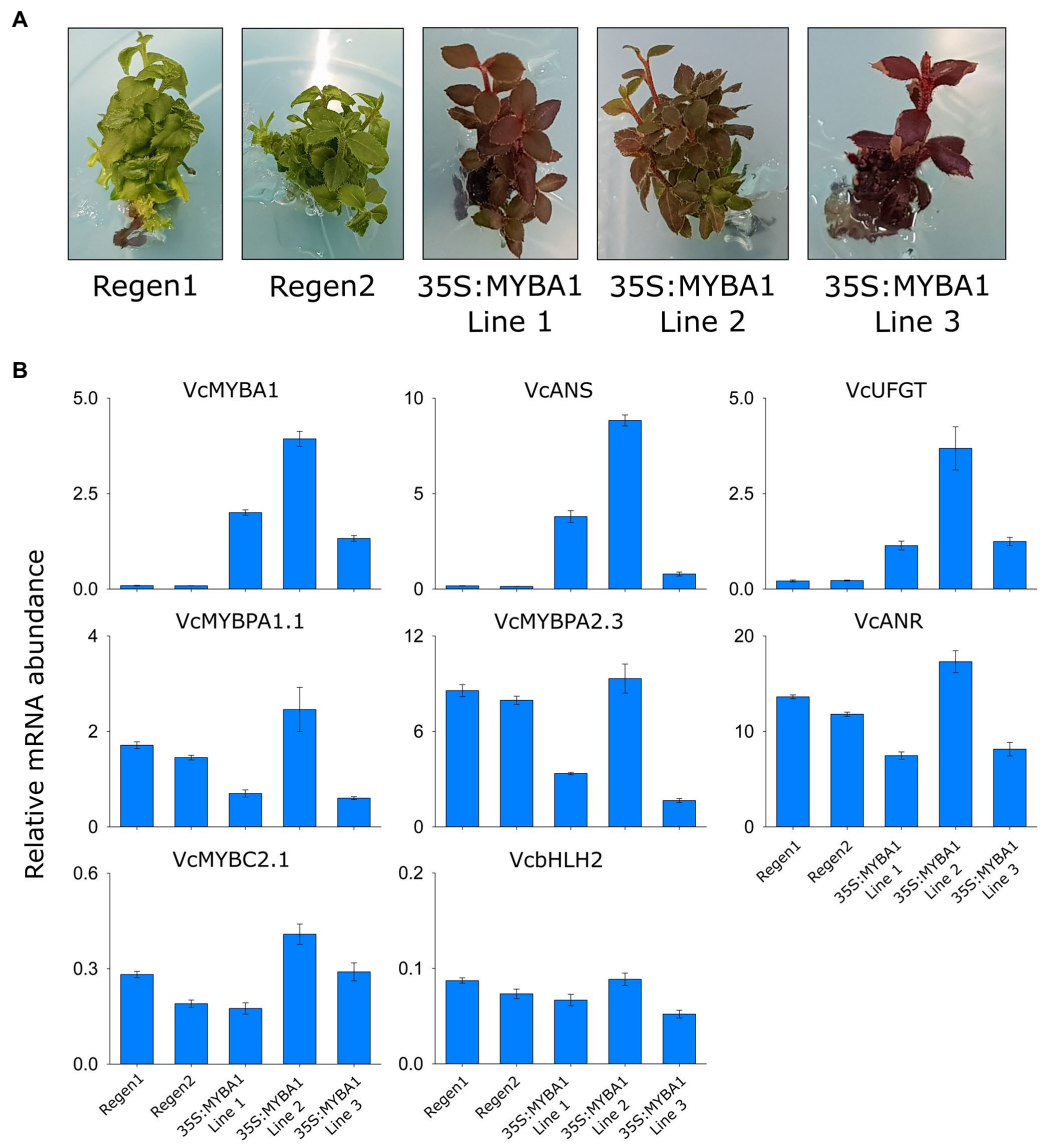
Stable overexpression of *VcMYBA1* in blueberry was sufficient to enhance vegetative anthocyanin content. **(A)** Pigmentation phenotype of two regeneration lines, Regen 1 and 2, and three *35S:VcMYBA1* lines in tissue culture. **(B)** Expression analysis was performed on the regeneration controls and *35S:VcMYBA1* lines using qRT-PCR. Values represent means ± SEs of three biological replicates. All values are relative to the geometric mean of abundance of *GAPDH* and *Actin* reference gene transcripts.

Evidence to date suggests that in addition to SG6/MYBA regulators, MYBPA1 and bHLH proteins are also required for regulating anthocyanins in bilberry and blueberry (Plunkett et al., 2018; Karppinen et al., 2021; Lafferty et al., 2022). To examine whether MYBA1 behaves as a high-level regulator within the hierarchy of TFs, we generated stable blueberry plants overexpressing *VcMYBA1* from a *CaMV35S* promoter. The regenerating transformed callus developed deep red anthocyanin pigmentation and gave rise to plants with dark red leaves, stems and roots (Figure 5A; Supplementary Figure S8). Activation of the anthocyanin biosynthesis genes *VcANS* and *VcUFGT* in the *35S:MYBA1* lines was confirmed by qRT-PCR, with minimal expression detected in the regeneration controls (Figure 5B). In contrast, the PA biosynthetic gene *ANR* and the regulators *MYBPA1.1* and *MYBPA2.3* were highly expressed in the regeneration controls, suggesting PAs were accumulating in these tissues. This was confirmed by staining leaves with DMACA, which revealed a strong accumulation of PAs in the regeneration controls (Supplementary Figure S9). The presence of high concentrations of anthocyanins in *35S:MYBA1* plants obscured detection of PAs with DMACA staining, despite clearing tissues with acetic acid/ethanol. In two independent *35S:MYBA1* lines (1 and 3), *MYBPA1.1* and *MYBPA2.3* expression was moderately reduced with a commensurate reduction in *ANR* transcripts (Figure 5B). The expression of *MYBC2.1* and bHLH2 were not greatly altered compared to controls.

## Discussion

Anthocyanin and PA biosynthesis in *Vaccinium* is complex, involving several known activator and repressor TFs, and potentially additional uncharacterized TFs. This study aimed to unravel the regulation of these pathways and identify the conserved elements of anthocyanin and PA regulation. We used differential expression and correlation analysis of blueberry and bilberry fruit developmental series to identify candidate anthocyanin-related TFs, and to determine the expression patterns of the flavonoid-related biosynthetic and TF genes during berry development. Stable transformation and functional analyses were then used to reveal a regulatory hierarchy between candidate TFs that facilitates strong activation of both pathways at different stages of berry development.

SG6 R2R3 MYBs are well-characterized anthocyanin regulators, and *MYBA1* is a key activator of anthocyanin biosynthesis in both blueberry and bilberry (Plunkett et al., 2018; Karppinen et al., 2021). Recently, a MYBPA1-type TF (*MYBPA1.1*) was shown to co-regulate the pathway and to be regulated by *MYBA1* (Karppinen et al., 2021; Lafferty et al., 2022). Both *MYBA1* and *MYBPA1.1* were strongly correlated with anthocyanin production and were expressed in bilberry red flesh, albeit at a lower concentration than in skin, while having minimal expression in pale blueberry flesh. These TFs correlated strongly with the anthocyanin specific biosynthetic genes *ANS* and *UFGT* in all anthocyanin-producing tissues (Supplementary Figure S4). Stable overexpression of *VcMYBA1* in blueberry was sufficient to elevate the anthocyanin content in vegetative tissues, with *ANS* and *UFGT* being highly upregulated (Figure 5). Interestingly, the leaves of the regeneration controls also contained high amounts of PAs, shown by DMACA staining, and the PA-specific regulator *MYBPA2.3* and biosynthesis gene *ANR* are highly expressed (Figure 5; Supplementary Figure S9). This finding was somewhat unexpected, but provides further support for the hierarchical regulation of TF genes (*bHLH2, MYBPA1.1, and MYBC2.1*) by MYBPA2.3 (Figure 4; Lafferty et al., 2022). However, this makes it very difficult to separate hierarchical regulation by *MYBA1* overexpression from the endogenous MYBPA2.3 action. It is anticipated that the *35S:MYBA1* blueberry plants will have additional phenotypes when they have been exflasked and reached maturity, which may include more intensely colored flowers and fruit with altered flesh color.

bHLH proteins are required for the formation of MBW complexes, and are themselves regulated by these complexes *via* a mechanism referred to as hierarchical regulation (Baudry et al., 2004, 2006; Albert et al., 2014). bHLH regulation has been reported by SG5 MYBs in Arabidopsis (Baudry et al., 2006; Xu et al., 2013), by SG6 MYBs in Arabidopsis, petunia, Antirrhinum and kiwifruit (Baudry et al., 2006; Xu et al., 2013; Albert et al., 2014, 2021; Liu et al., 2021) and by MYBPA1-type MYBs in grape (Hichri et al., 2010). *VcbHLH2,* previously identified as a potential PA and anthocyanin regulator in blueberry (Günther et al., 2020; Lafferty et al., 2022), was found to correlate with anthocyanin production and biosynthetic gene activity in tissues containing high concentrations of anthocyanins (Figure 1C; Supplementary Figure S5). The *VcbHLH2* promoter was activated by the SG5 *VcMYBPA2.3,* SG6 *VcMYBA1* and MYBPA1-type *VcMYBPA1.1* activators, revealing that *bHLH2* was also hierarchically regulated in *Vaccinium* (Figure 4). Hierarchical regulation of *bHLH2* by MYBPA2.3 explains why *bHLH2* is expressed in wild type blueberry leaves (Figure 4); MYBPA2.3 is highly expressed and regulates PA biosynthesis, *bHLH2* and other TF targets (see below). Interestingly, overexpression of *MYBA1* was unable to increase *VcbHLH2* expression any further (Figure 5).

MYB repressors have previously been proposed to fine-tune the anthocyanin and PA pathways, preventing over-accumulation of these metabolites (Albert et al., 2014; Albert, 2015). In this study, the R3 MYB, *MYBR3.1*, was expressed during early berry development, when PAs are accumulating, and at ripening, when anthocyanins are produced (Figure 2A), and functional assays demonstrate VcMYBR3.1 inhibits the production of both metabolites (Figure 2B). This suggests that R3-MYB repressors are also important for regulating these pathways in *Vaccinium* berries, as they are in other characterized systems (Schellmann et al., 2002; Zhu et al., 2009; Albert et al., 2014; Cao et al., 2017; Colanero et al., 2018; Ding et al., 2020).

MYBR3.1 has repressive activity upon anthocyanin and PA biosynthesis that is dependent on bHLH concentration. The addition of *PpbHLH3* in promoter activation assays removed its repression activity, while doubling the amount of *VcMYBR3.1* in infiltrations increased the inhibitory effect on activator MYBs (Figure 2C). This is consistent with the proposed role of R3 MYB repressors titrating bHLH proteins (Zhu et al., 2009; Albert et al., 2014). The activator MYBs assayed (*MYBA, MYBPA1, MYBPA2*) were probably able to function without the addition of *PpbHLH3* because of the presence of endogenous *N. benthamiana* bHLHs (Montefiori et al., 2015). R3 MYB repressors can also be hierarchically regulated by activator MYBs, as reported in petunia and tomato (Albert et al., 2014; Yan et al., 2020). This was, however, not seen for the *VcMYBR3.1* promoter assayed in this study. Additionally, *MYBR3.1* had similar expression in blueberry and bilberry flesh, regardless of MYB activators having minimal expression in blueberry flesh. This indicated an additional (MYBA1-independent) regulatory mechanism regulates *MYBR3.1* expression in blueberry flesh at ripening. This is further supported by the analysis of an albino bilberry mutant, in which *VmMYBR3* had elevated expression (Zorenc et al., 2017), where *VmMYBA1* expression is expected to be minimal.

**Figure 6:**
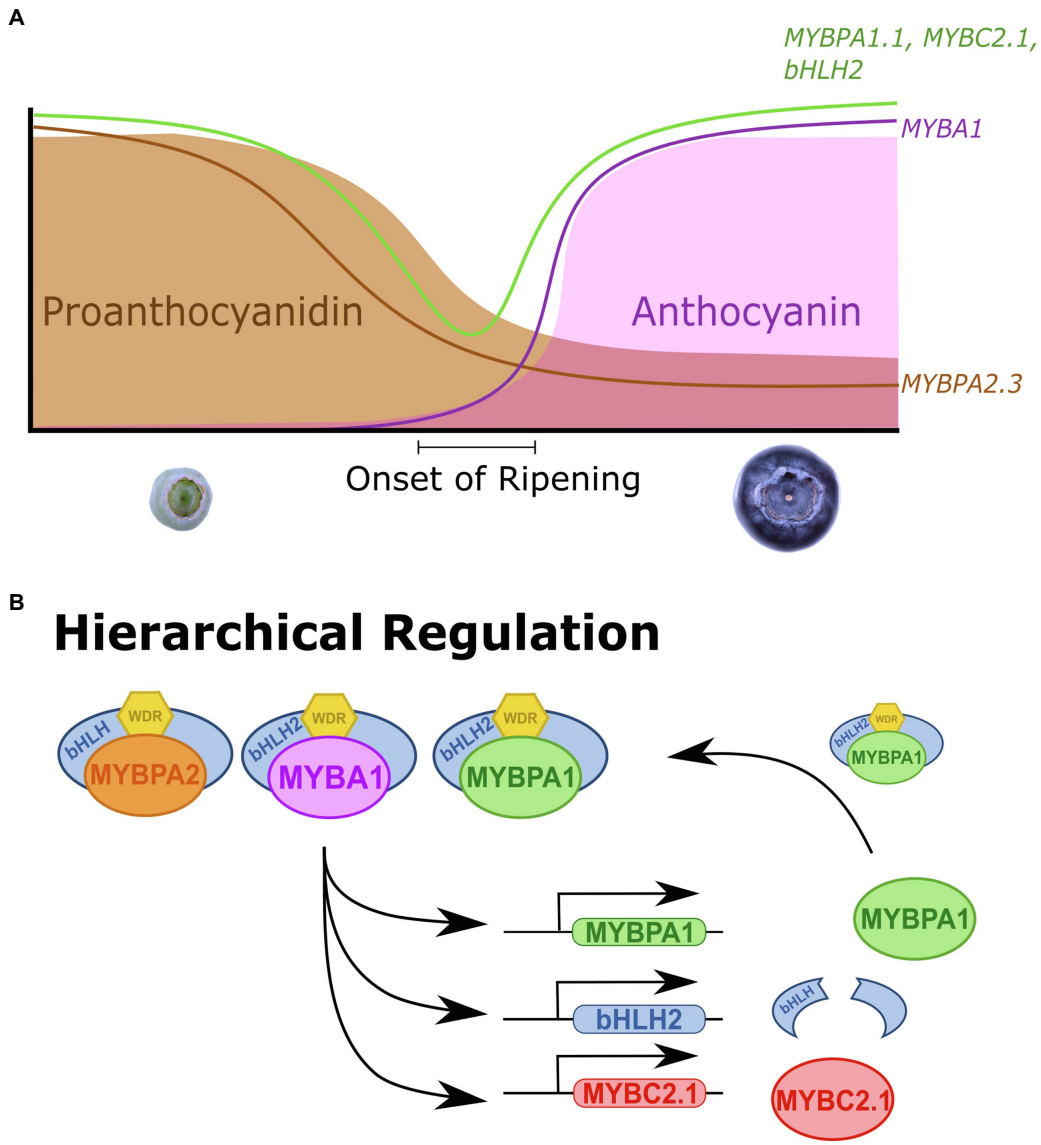
*Vaccinium* MYB activators and repressors are coordinately regulated for precise control of proanthocyanidin and anthocyanin biosynthesis. **(A)** During early stages of *Vaccinium* fruit development (S1–S3) proanthocyanidin (PA) concentrations are high, before declining as development proceeds. This correlates with the expression of the *MYBPA2.3*, a PA-specific SG5 MYB activator. At the onset of ripening (S5) anthocyanins begin accumulating and concentrations rise as ripening progresses, correlating with the anthocyanin-specific SG6 MYB, *MYBA1*. *MYBPA1.1, MYBC2.1, MYBR3.1,* and *bHLH2* regulate both PA and anthocyanin biosynthesis and this was reflected in their biphasic expression pattern, being more strongly expressed during early development and again during ripening. **(B)** SG5 MYBPA2, SG6 MYBA1 and MYBPA1-type MYBPA1 MBW complexes hierarchically regulate *MYBPA1, bHLH2,* and *MYBC2.1*. This establishes feed forward activation for *bHLH2* and *MYBPA1* and a feedback repression loop for *MYBC2.1*.

*VcMYBC2.1* encodes a SG4 MYB and was identified in the expression analyses that was expressed highly during early development and ripening, indicating it was a repressor of both PA and anthocyanin production. *VcMYBC2.1* correlated strongly with anthocyanin biosynthesis and *UFGT* in anthocyanin rich tissues (Figure 1C; Supplementary Figure S5). The anthocyanin and PA accumulation and promoter activation assays confirmed that *VcMYBC2.1* was a repressor of both pathways (Figure 3). In other plant species, expression of the *SG4 MYB* genes correlated with the pathways they repress, because they are themselves regulated by SG5 and/or SG6 MYBs (Albert et al., 2014; Jun et al., 2015; Yoshida et al., 2015; Huang et al., 2019). The hierarchical regulation by both SG5 and SG6 MYBs was found in *Vaccinium* (Figure 6), supporting this as a conserved aspect of anthocyanin and PA pathway regulation in different tissues across eudicots.

Hierarchical activation of *MYBPA1* by SG5 MYB activators has been reported in grape, poplar, apple and blueberry (Terrier et al., 2009; James et al., 2017; Wang et al., 2018; Lafferty et al., 2022) and activation by SG6 MYB activators has recently been shown in blueberry (Lafferty et al., 2022). Furthermore, MYBPA1 proteins have been shown to activate the promoters of many general flavonoid biosynthetic genes leading to PA and anthocyanin production (Bogs et al., 2007; Ravaglia et al., 2013; Karppinen et al., 2021; Lafferty et al., 2022). However, the position of the different TF types in the wider context of flavonoid production and hierarchical regulation has not been elucidated. Here we show that VcMYBPA1.1 could activate the *VcbHLH2* promoter, contributing to the feedforward activation loop. Additionally, *VcMYBPA1.1* could activate the *VcMYBC2.1* promoter as part of a feedback repression loop. This establishes MYBPA1 proteins as important hierarchical regulators in the wider PA and anthocyanin regulatory modules, expanding the models proposed by Albert et al. (2014) of hierarchical regulation within the MBW complex.

Our results underpin a proposed model that highlights the complex nature of *Vaccinium* anthocyanin and PA regulation (Figure 6). During early fruit development PA metabolites are produced in unripe fruit, deterring herbivory when seeds are not mature (Jaakola et al., 2002; Zifkin et al., 2012). The PA specific SG5 *MYBPA2* activators are expressed, which hierarchically regulates the *MYBPA1* (Lafferty et al., 2022) and *bHLH2* activators. This functions as a feed forward activation loop, providing additional components for MBW complex formation. The activators also regulate the SG4 MYB repressor *MYBC2.1,* establishing a feedback repression loop (Albert et al., 2014), which limits the activation of the pathway. *MYBR3.1* also represses the PA pathway, although how it fits into the regulatory system is unresolved. PA concentration and *MYBPA2* expression fall as development proceeds. Anthocyanin content rises at the onset of ripening (Jaakola et al., 2002; Zifkin et al., 2012). This coincides with a rise in expression of the anthocyanin-specific SG6 *MYBA1* TF. The expression of *MYBPA1* and *bHLH2* activators and the *MYBC2.1* repressor also increase, concurrent with the feedforward activation and feedback repression loops regulating anthocyanin biosynthesis, along with *MYBR3.1*. The lack of anthocyanin content in blueberry flesh is probably due to the minimal expression of *MYBA1*. Target TF and biosynthetic genes also have minimal expression and the anthocyanin pathway was unable to be activated. It remains to be seen if MYBA1 is indeed the limiting factor for flesh color, but our transgenic results show strong anthocyanin accumulation in all vegetative tissues with the overexpression of MYBA and, based on data from other species, it is likely that this will extend to flesh colors (Espley et al., 2007; Lin-Wang et al., 2014; Rinaldo et al., 2015; Hijaz et al., 2018).

In conclusion, transcriptomic analysis in fruit skin and flesh samples across blueberry and bilberry development identified a number of TFs that strongly correlate with anthocyanin pigmentation in ripening blueberry skin and bilberry skin and flesh. These included two distinct MYB repressors, *MYBC2.1* and *MYBR3.1*, which were functionally characterized. Promoter activation assays revealed MYBPA1 as an important hierarchical regulator for PA and anthocyanin regulation, being able to activate *VcbHLH2* and *VcMYBC2.1*. These TFs were also activated by the SG5 MYBPA2 and SG6 MYBA1 proteins. This confirmed that regulation of PA and anthocyanin biosynthesis is complex, requiring coordinated expression of both activator and repressor TFs. Furthermore, the overexpression of *MYBA1* was sufficient for strong activation of the anthocyanin pathway in transgenic blueberry plants. In the transcriptomic analyses *MYBA1* was expressed in bilberry skin and flesh and blueberry skin, with minimal expression in blueberry flesh. Based on these results we conclude that the expression of *MYBA1* in bilberry flesh is probably the crucial determinant of red flesh.

## Data Availability Statement

The datasets presented in this study can be found in online repositories. The names of the repository/repositories and accession number(s) can be found at: https://www.ncbi.nlm.nih.gov/, with NCBI accessions PRJNA591663 and PRJNA739815.

## Author Contributions

DJL: performed experimental work and data analysis; CHD and CSG: provided bioinformatic support; LJ and KK: providing berry samples; MB, LW, and HL: produced transgenic lines; NWA, APD, RVE, and ACA: conceptualization. All authors contributed to the article and approved the submitted version.

## Funding

This study was supported by the New Zealand Ministry of Business, Innovation, and Employment contract C11X1704 “Filling the Void: boosting the nutritional content of New Zealand fruit.”

## Conflict of Interest

Authors DJL, RVE, CHD, APD, CSG, MB, LW, ACA, and NWA were employed by the New Zealand Institute for Plant and Food Research Limited.

The remaining authors declare that the research was conducted in the absence of any commercial or financial relationships that could be construed as a potential conflict of interest.

## Publisher’s Note

All claims expressed in this article are solely those of the authors and do not necessarily represent those of their affiliated organizations, or those of the publisher, the editors and the reviewers. Any product that may be evaluated in this article, or claim that may be made by its manufacturer, is not guaranteed or endorsed by the publisher.

## Abbreviations

PA: Proanthocyanidin
TF: Transcription factor
CHS: Chalcone synthase
F3′5′ H: Flavonoid 3′5′ *-*hydroxylase
DFR: Dihydroflavonol 4-reductase
ANS: Anthocyanidin synthase
UFGT: UDP-glucose:flavonoid 3-*O*-glucosyltransferase
GST: Glutathione *S*-transferase
MATE: Multidrug and Toxic Compound Extrusion
ANR: Anthocyanidin reductase
LAR: Leucoanthocyanidin reductase
FLS: Flavonol synthase
MYB: Myeloblastosis
bHLH: Basic helix loop helix.
