# Supplementary material for: The Coordinated Action of MYB Activators and Repressors Controls Proanthocyanidin and Anthocyanin Biosynthesis in Vaccinium
Source: Front Plant Sci. 2022 Jun 24;13:910155. doi: 10.3389/fpls.2022.910155 (PMC9263919; doi:10.3389/fpls.2022.910155)
Supplement: Supplementary file 1 [file Data_Sheet_1.PDF]

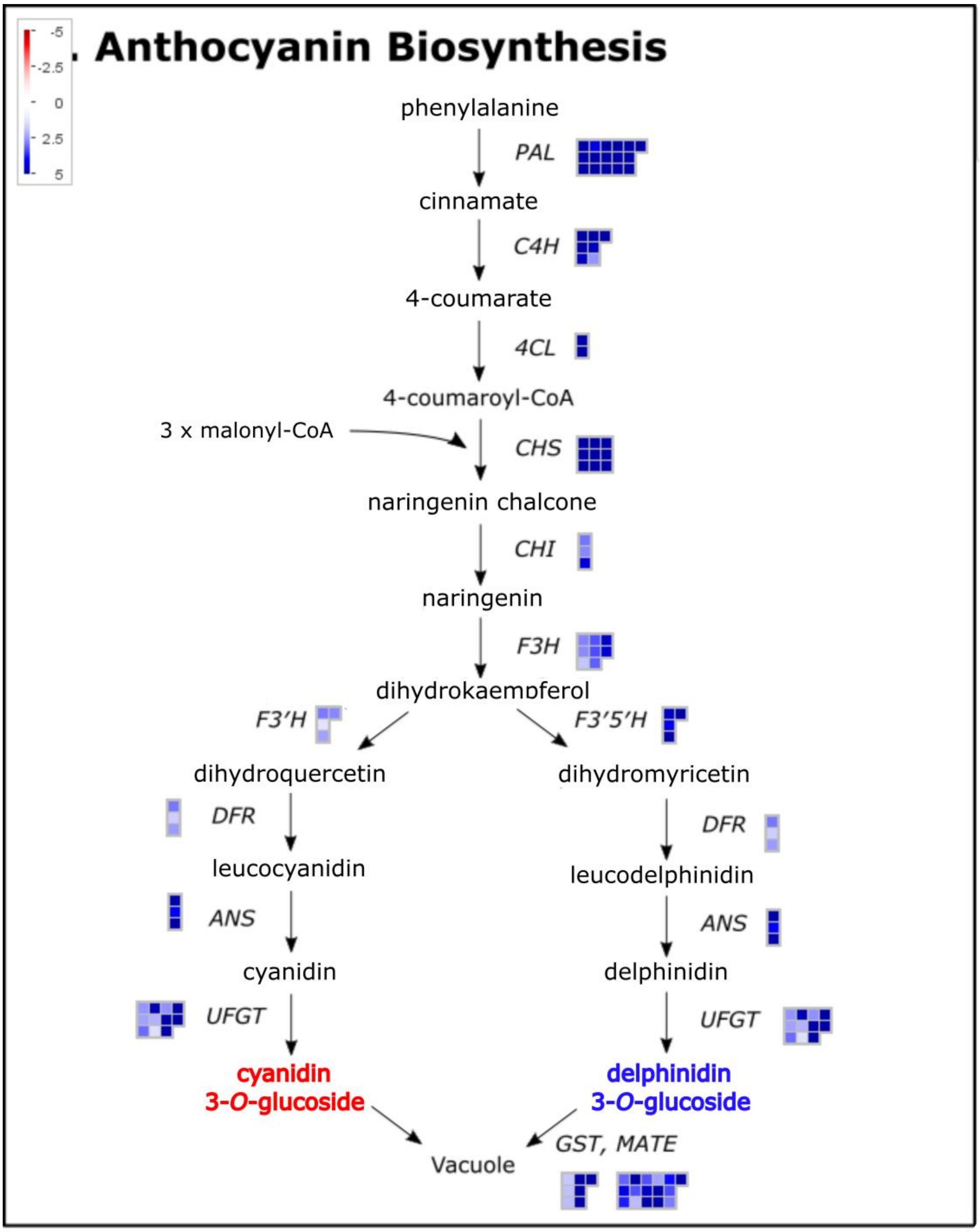

Supplementary Figure 1 Flavonoid biosynthetic genes are significantly highly expressed in bilberry fruit flesh compared to blueberry flesh

Differential gene expression data for biosynthetic genes leading to anthocyanin biosynthesis, between blueberry and bilberry S7 flesh, was visualised using the Mapman software (V3.5.1R2). Each box represents a gene ID and the colour displays the log2 fold change, with blue and red being either upregulated or downregulated in bilberry, respectively. The pathway depicts the biosynthesis of the major anthocyanin classes found in blueberry and bilberry fruit (pelargonidin-based anthocyanins are not produced).

A

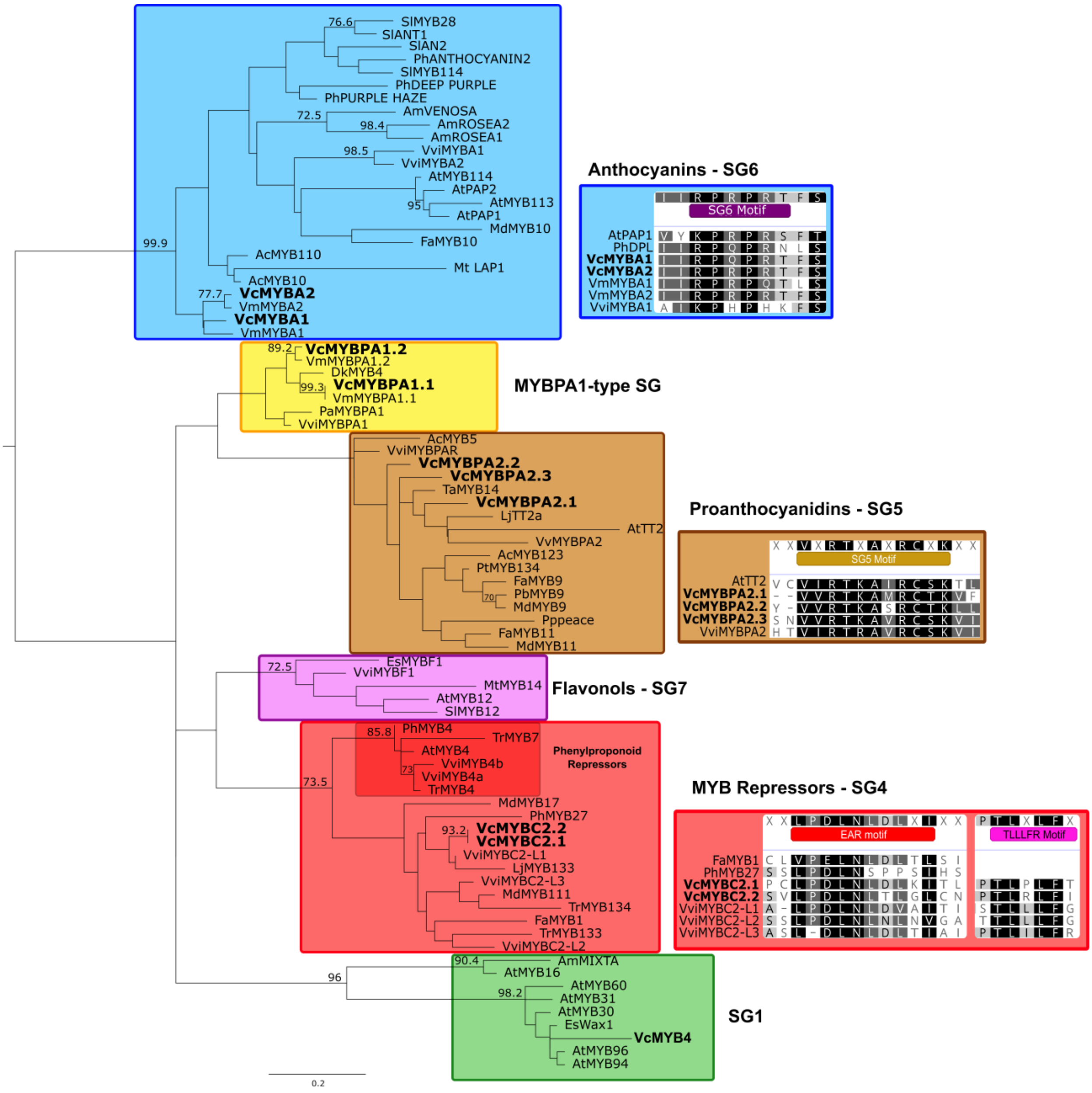

SlMYB28
SlANT1
SlAN2
PhANTHOCYANIN2
SlMYB114
PhDEEP PURPLE
PhPURPLE HAZE
AmVENOSA
AmROSEA2
AmROSEA1
VviMYBA1
VviMYBA2
AtMYB114
AtPAP2
AtMYB113
AtPAP1
MdMYB10
FaMYB10
AcMYB110
Mt LAP1
AcMYB10
VcMYBA2
VmMYBA2
VcMYBA1
VmMYBA1
Anthocyanins - SG6
SG6 Motif
AtPAP1
PhDPL
VcMYBA1
VcMYBA2
VmMYBA1
VmMYBA2
VviMYBA1
VcMYBPA1.2
VmMYBPA1.2
DkMYB4
VcMYBPA1.1
VmMYBPA1.1
PaMYBPA1
VviMYBPA1
MYBPA1-type SG
AcMYB5
VviMYBPAR
VcMYBPA2.2
VcMYBPA2.3
TaMYB14
VcMYBPA2.1
LjTT2a
AtTT2
VvMYBPA2
AcMYB123
PtMYB134
FaMYB9
PbMYB9
MdMYB9
Pppeace
FaMYB11
MdMYB11
Proanthocyanidins - SG5
SG5 Motif
AtTT2
VcMYBPA2.1
VcMYBPA2.2
VcMYBPA2.3
VviMYBPA2
EsMYBF1
VviMYBF1
MtMYB14
AtMYB12
SlMYB12
Flavonols - SG7
PhMYB4
TrMYB7
AtMYB4
VviMYB4b
VviMYB4a
TrMYB4
Phenylproponoid Repressors
MdMYB17
PhMYB27
VcMYBC2.2
VcMYBC2.1
VviMYBC2-L1
LjMYB133
VviMYBC2-L3
MdMYB111
TrMYB134
FaMYB1
TrMYB133
VviMYBC2-L2
MYB Repressors - SG4
EAR motif
TLLLFR Motif
FaMYB1
PhMYB27
VcMYBC2.1
VcMYBC2.2
VviMYBC2-L1
VviMYBC2-L2
VviMYBC2-L3
AmMIXTA
AtMYB16
AtMYB60
AtMYB31
AtMYB30
EsWax1
VcMYB4
AtMYB96
AtMYB94
SG1
0.2

B

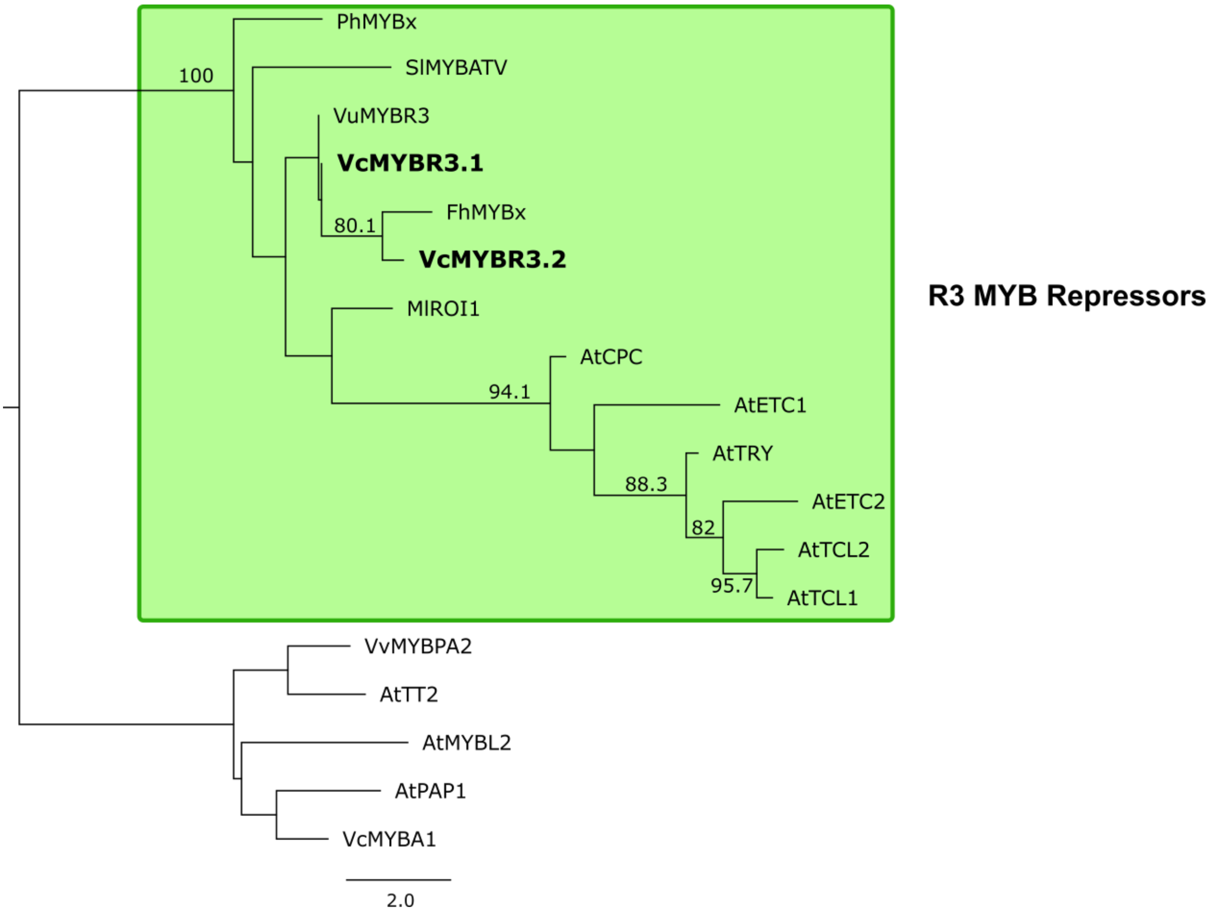

Supplementary Figure 2 Phylogenetic analysis of candidate MYB TFs

A maximum likelihood phylogenetic tree of (A) R2R3 and (B) R3 MYB proteins was constructed using PhyML (Guindon et al., 2010). For the R2R3 MYB tree, the deduced amino acid sequences were trimmed to only include the R2R3 MYB domain. Sequences were aligned using the Geneious alignment (Kearse et al., 2012). Node support > 70% from 1000 bootstrap replicates is shown. For the R3 MYB tree, R2R3 MYBs were used as an outgroup. Sequences from *Vaccinium corymbosum* are in bold. Insets show motifs specific to the given subgroup. *V. corymbosum* MYBs were aligned, using Geneious alignment (Kearse et al., 2012) with representative genes from that subgroup.

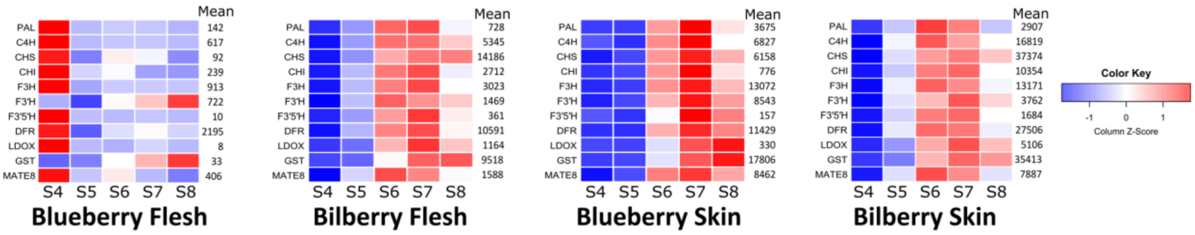

Supplementary Figure 3 Visualisation of additional biosynthetic genes' expression.

The developmental expression patterns of additional biosynthetic genes was visualised as a heatmap. Raw counts were normalized via the median of ratios method (Love et al., 2014). Count data was visualized as Z-scores, which calculated how many standard deviations a data point was from the mean of a gene across all samples in the given tissue, with blue and red representing negative and positive Z-scores, respectively. Mean represented the mean counts for each gene, in each tissue type, across development.

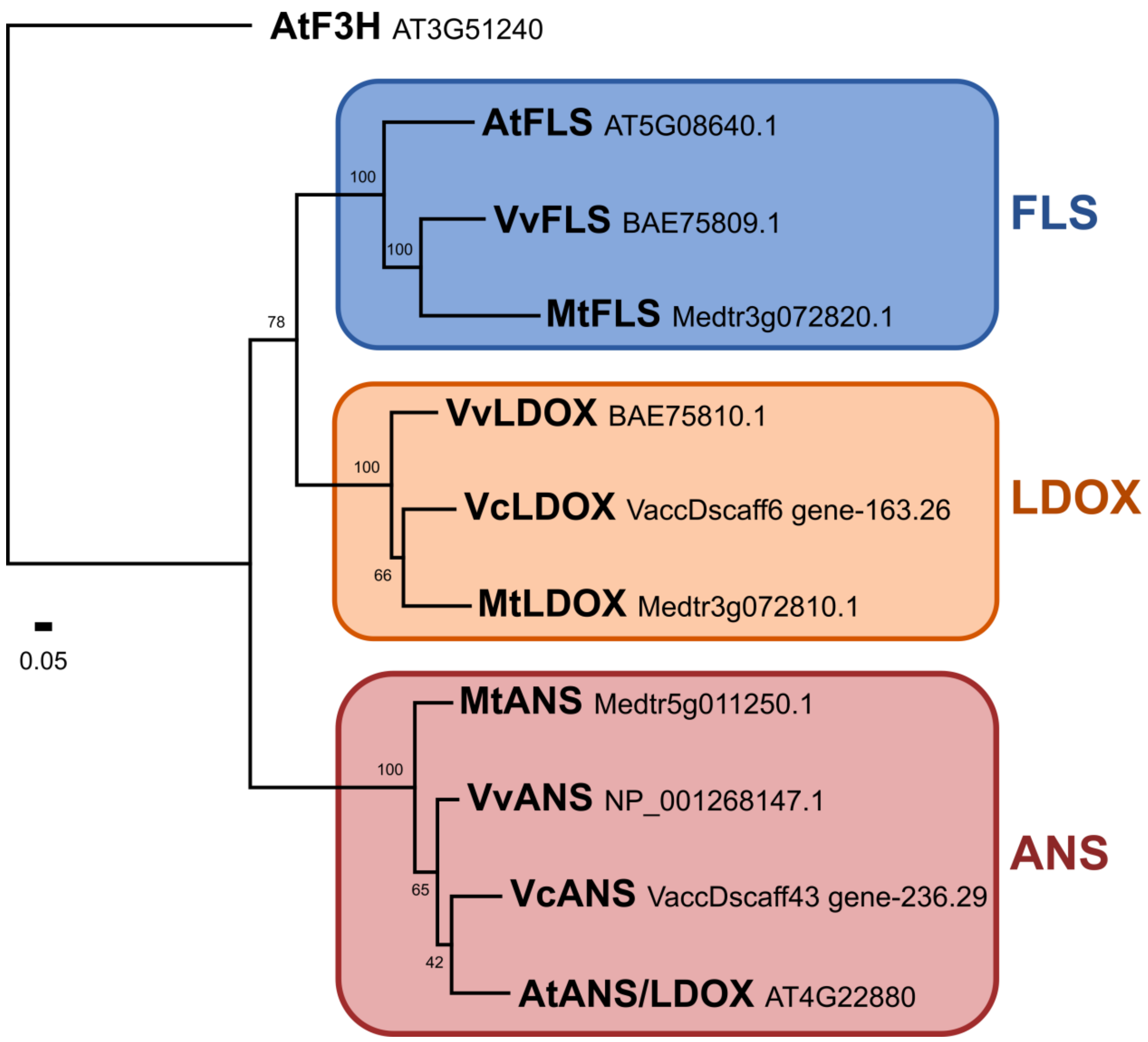

Supplementary Figure 4 Phylogenetic tree of ANS and LDOX proteins

A maximum likelihood phylogenetic tree was constructed based on an amino acid alignment (MUSCLE) of selected flavonoid 2-oxoglutarate dependent dioxygenase proteins. F3H flavanone 3-hydroxylase, FLS flavonol synthase, LDOX leucoanthocyanidin dioxygenase, ANS anthocyanidin synthase. Bootstrap values from 1000 replicates are shown. Genbank sequence IDs or gene models are indicated. At *Arabidopsis thaliana*, Mt *Medicago truncatula*, Vc *Vaccinium corymbosum*, Vv *Vitis vinifera*.

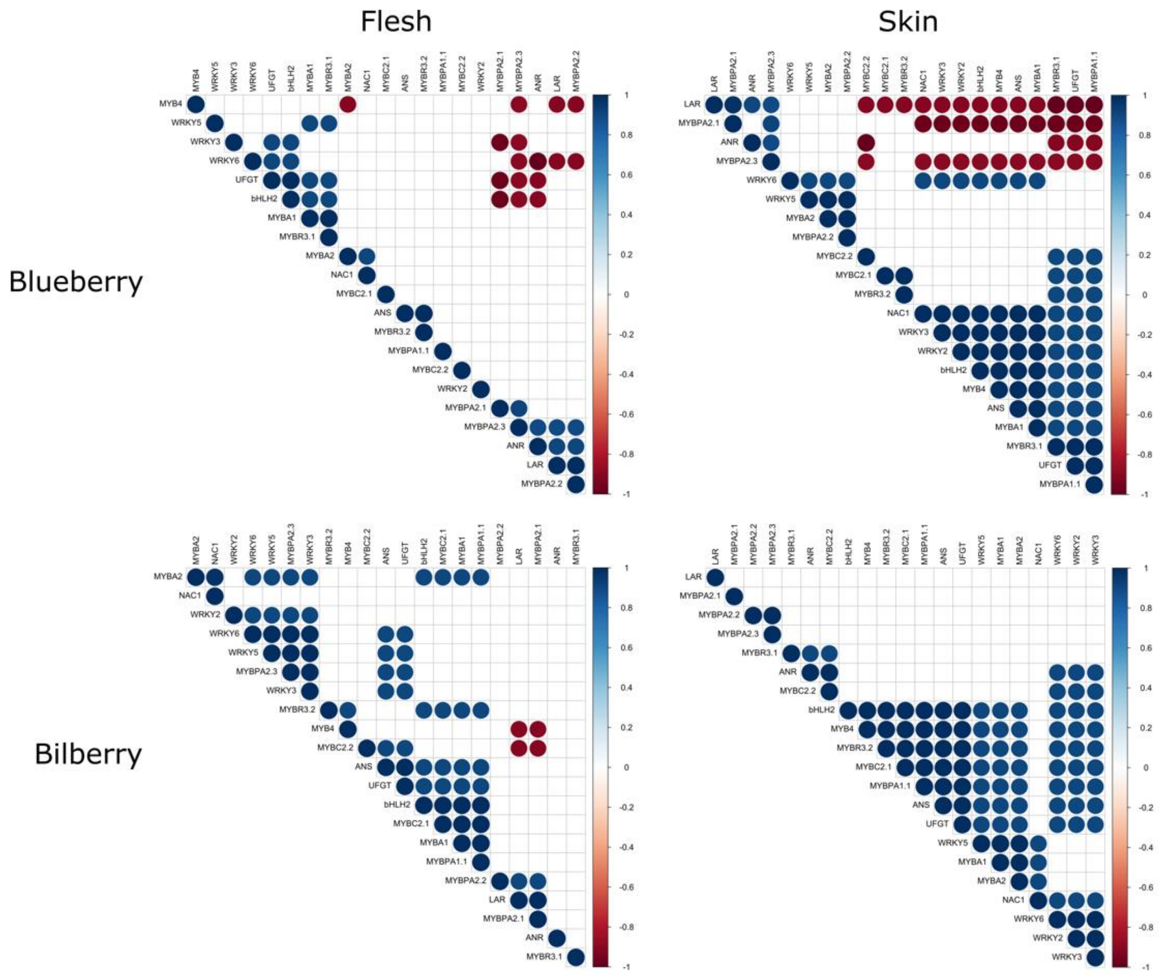

Supplementary Figure 5 Correlation analysis revealed candidate TFs that strongly correlated with characterised anthocyanin regulators and biosynthetic genes

Correlation analysis was performed on the normalised RNA-seq count data of selected flavonoid biosynthetic genes and candidate TFs using Spearman rank correlations. Each tissue type was analysed separately. Blue and red circles indicate significant positive and negative correlations (α = 0.05), respectively. Non-significant correlations remain blank.

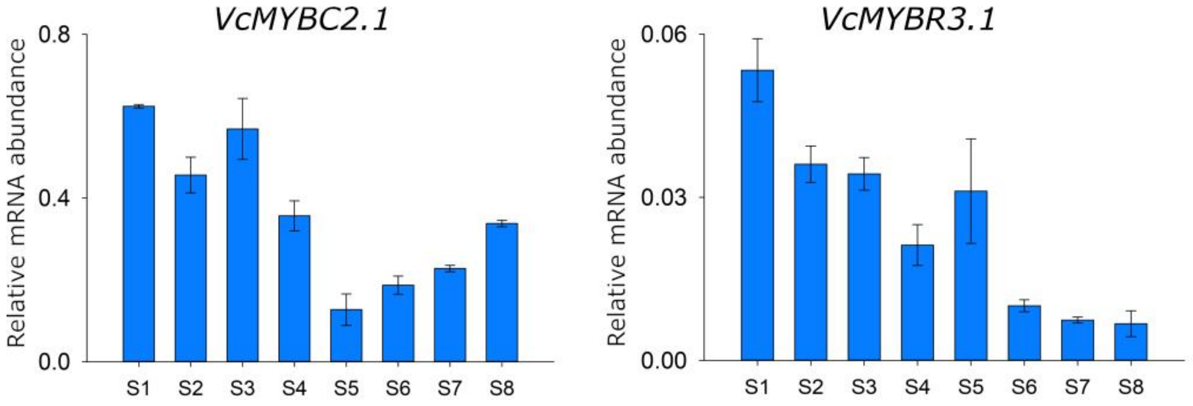

Supplementary Figure 6 Expression of VcMYBC2.1 and VcMYBR3.1 during blueberry fruit development

Transcript abundance of *VcMYBC2.1* and *VcMYBR3.1* across a full blueberry 'Nui' fruit development series, determined by qRT-PCR. Values represent means ± SEs of three biological replicates. All values are relative to the geometric mean of abundance of GAPDH and Actin reference gene transcripts.

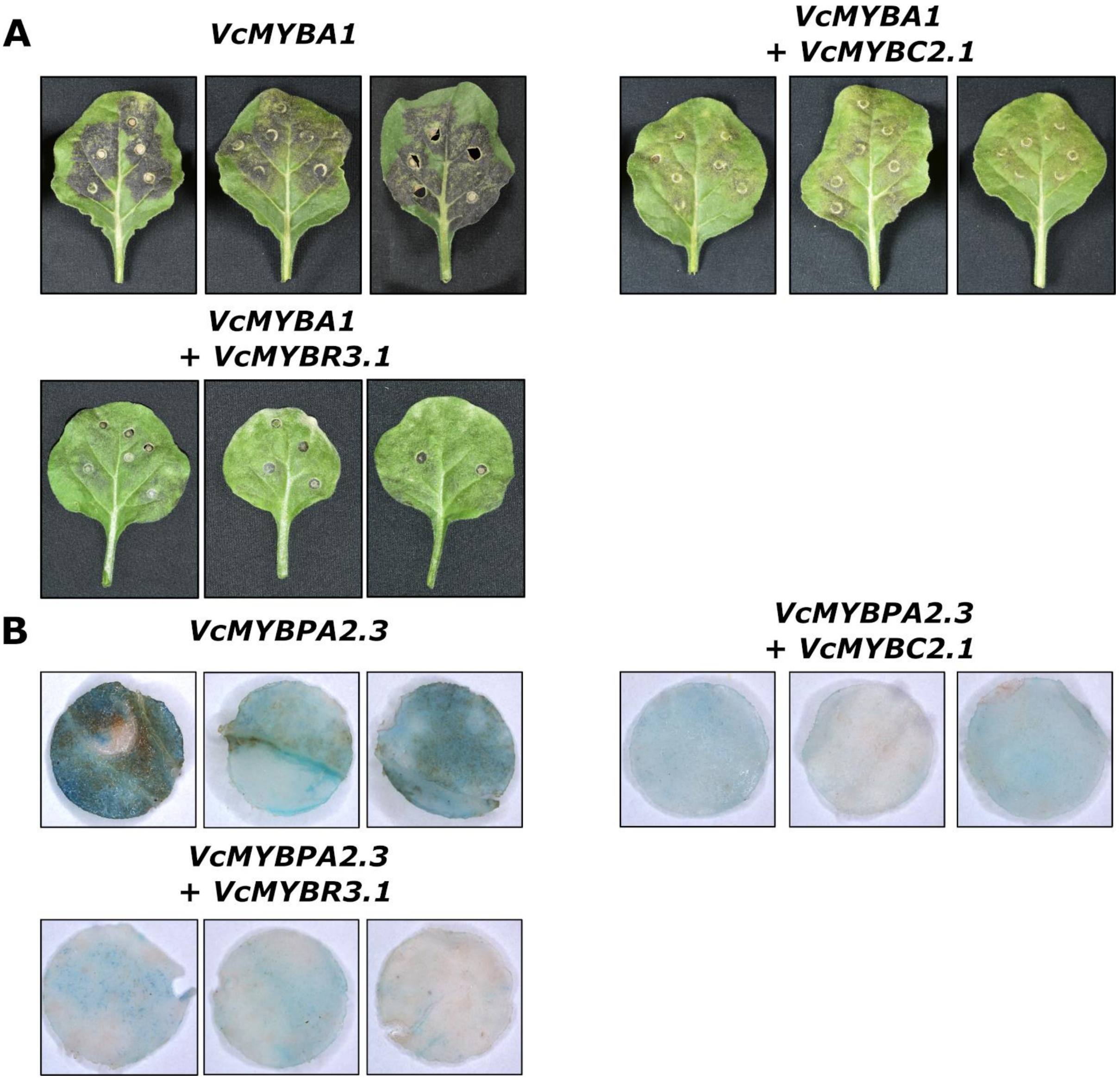

Supplementary Figure 7 Anthocyanin and PA accumulation phenotypes of *Nicotiana benthamiana* plants after transient overexpression of activator and repressor MYBs

A**.** Anthocyanin accumulation phenotypes of *N. benthamiana* leaves 5 days co-infiltrated with *MYBA1* and *PpbHLH3*, with and without MYB repressors (*VcMYBC2.1*, *VcMYBR3.1*), in overexpression constructs. Three leaves were infiltrated per treatment, as biological replicates. B. Proanthocyanidin accumulation phenotypes of *N. benthamiana* leaves 5 days post infiltration, co-infiltrated with *MYBPA2.3* and *PpbHLH3*, with and without MYB repressors, in overexpression constructs. Proanthocyanidins were visualised by DMACA-HCl staining, showing reduced intensity of blue DMACA staining when *VcMYBC2.1* or *MYBR3.1* were included compared with VcMYBPA2.3 alone. Leaf discs, 1 cm diameter, were taken and DMACA stained before being photographed. Three leaves were infiltrated per treatment, as biological replicates.

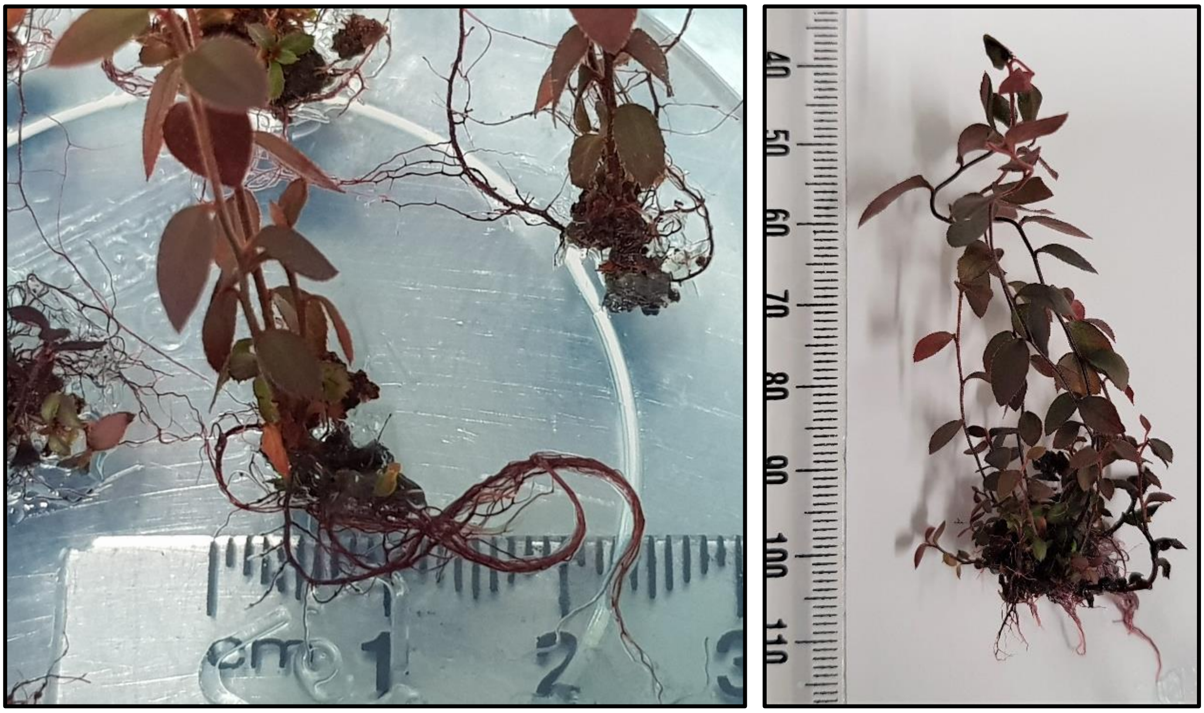

Supplementary Figure 8 Pigmentation phenotypes of *35S:VcMYBA1* transgenic blueberry plants in tissue culture

Photographs of blueberry plants, in tissue culture, overexpressing *VcMYBA1* were taken to show the pigmentation phenotypes of the various vegetative tissue types. A ruler was included to show the size of the plants.

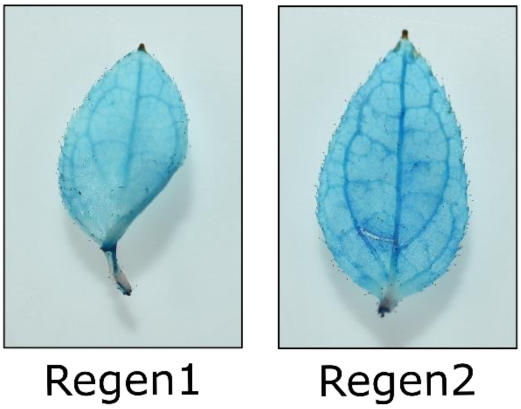

Supplementary Figure 9 DMACA staining of leaves from regeneration controls

Leaves from the regeneration controls were DMACA stained, to show the presence of proanthocyanidins, and photographed
