# Supplementary material for: The Coordinated Action of MYB Activators and Repressors Controls Proanthocyanidin and Anthocyanin Biosynthesis in Vaccinium
Source: Front Plant Sci. 2022 Jun 24;13:910155. doi: 10.3389/fpls.2022.910155 (PMC9263919; doi:10.3389/fpls.2022.910155)
Supplement: Supplementary file 2 [file Data_Sheet_2.docx]

**Supplemental Table S2.** ‘Draper’ genome gene IDs and genbank numbers

| **Gene** | **‘Draper’ gene ID** | **Genbank** |
| --- | --- | --- |
| *CHS* | augustus_masked-VaccDscaff9-processed-gene-64.0 | JN654702.1 |
| *F3’5’H* | maker-VaccDscaff8-augustus-gene-360.23-mRNA-1 | MH321464.1 |
| *DFR* | augustus_masked-VaccDscaff13-processed-gene-166.8 | KF960989 |
| *ANS* | maker-VaccDscaff43-augustus-gene-236.29 | JN654701.1 |
| *UFGT* | maker-VaccDscaff6-augustus-gene-420.36 | MH321467.1 |
| *ANR* | maker-VaccDscaff19-augustus-gene-256.19 | MH321471.1 |
| *LAR* | maker-VaccDscaff9-augustus-gene-315.13 | MH321470.1 |
| *MYBA1* | maker-VaccDscaff1486-snap-gene-0.3 | MH105054 |
| *MYBA2* | maker-VaccDscaff13-augustus-gene-105.26-mRNA-1 |  |
| *MYBPA1.1* | maker-VaccDscaff39-snap-gene-168.25 | JQ085966 |
| *MYBPA2.1* | maker-VaccDscaff32-augustus-gene-55.27 |  |
| *MYBPA2.2* | maker-VaccDscaff34-augustus-gene-10.31 |  |
| *MYBPA2.3* | maker-VaccDscaff33-snap-gene-307.38 |  |
| *MYBC2.1* | maker-VaccDscaff28-augustus-gene-197.19 |  |
| *MYBC2.2* | maker-VaccDscaff4-augustus-gene-330.20 |  |
| *MYBR3.1* | maker-VaccDscaff4-snap-gene-174.23 |  |
| *MYBR3.2* | maker-VaccDscaff1069-augustus-gene-0.8 |  |
| *WRKY2* | maker-VaccDscaff26-augustus-gene-44.51 |  |
| *WRKY3* | maker-VaccDscaff11-augustus-gene-207.26 |  |
| *WRKY5* | maker-VaccDscaff4-augustus-gene-398.15 |  |
| *WRKY6* | maker-VaccDscaff19-augustus-gene-368.29 |  |
| *NAC1* | maker-VaccDscaff30-augustus-gene-305.45 |  |
| *bHLH2* | maker-VaccDscaff19-augustus-gene-381.30 |  |
| *MATE8* | maker-VaccDscaff4-snap-gene-174.23 | KF875439.1 |
| *PAL* | maker-VaccDscaff10-augustus-gene-222.29 |  |
| *C4H* | augustus_masked-VaccDscaff33-processed-gene-307.8 |  |
| *CHI* | maker-vaccdscaff26-snap-gene-196.29 | MH321461.1 |
| *F3H* | maker-VaccDscaff32-augustus-gene-323.35 |  |
| *DFR* | augustus_masked-VaccDscaff13-processed-gene-166.8 | KF960989 |
| *LDOX* | maker-VaccDscaff6-augustus-gene-163.26 |  |
| *GST* | maker-VaccDscaff30-augustus-gene-282.25 |  |

| Gene | Forward primer (5'-3') | Reverse primer (5'-3') |
| --- | --- | --- |
| *VcMYBR3.1* | CACCAAAATGGCTGACTCAGAACAC (TOPO) | TCATTCGCTCGTTGAGTATCTGGAG |
| *VcMYBC2.1* | CACCAAAATGAGGAAGCCATGTTGTG (TOPO) | TCATGTAAAGAGAGGAAGGGTG |
| *VcMYBR3.1pro* | ATTGCTCGAGTGTATGGATAGGTACATGAGTAGGA (XhoI) | TTACGAATTCTTGGAGAGAGGGGGAGAG (EcoRI) |
| *VcMYBC2.1pro* | GGATCTGCAGGTGGAGTCTGTGCACTCCT (Pst I) | GTACCATGGTCTACTCACACAGAGTACATAAACCTC (NcoI) |
| *VcbHLH2pro* | attgGCGGCCGCTCTCACTATGTTTTGTTTGTGTAGGCTAGA (NotI) | gtaCCATGGCTCCGCCGCTGGCCAC (NcoI) |

**Supplemental Table S3.** Primers used for isolating and cloning sequences into vectors for functional analysis

| Gene | Forward primer (5'-3') | Reverse primer (5'-3') |
| --- | --- | --- |
| *VcANS* | TTCCACACTGTTTACCCTGAGG | CTTCTTCCAATCCCAAGCAGAC |
| *VcANR* | CCGACAATCACAAGAAGACC | GGTCACAACCTGTTATTGGG |
| *VcUFGT* | GGAAGATTGGTGTGAGAGTGG | TGTTCCCTCAATTCCTTCCCC |
| *VcbHLH2* | TAGAGAGTGATGCGTTGG | CATTAGTCAACGACGACTGC |
| *VcMYBA1* | CAACCCATCCCAAACACAACC | CCACGTCATCATACCTCTGC |
| *VcMYBPA1.1* | TCCTAACGACCAACCACCA | GGGCTTTGGGTTATGGACTT |
| *VcMYBPA2.3* | TCCACAGCCAAACATGAAAA | CCAAAGCAAAATCCCTTGAG |
| *VcMYBC2.1* | AGGAGCATGGTCCAAACAAG | TTCCCACAACGAAGTAGTCC |
| *VcMYBR3.1* | GAGCGAATTCAGGCATCTGT | CCCAAAAACCTTGAACACGA |

**Supplemental Table S4.** Blueberry gene-specific primers used for RT-qPCR analysis

**Supplemental Table S5. Differentially expressed TFs identified in DEseq2 analysis.**

Mapman annotaton is shown for each gene ID, with gene IDs representing the same gene grouped. The amino acid sequence of the gene IDs were BLASTP queried against the Araport11 protein sequences dataset using TAIR BLAST 2.9.0+ and the Arabidopsis accession number and annotation of the best hit is displayed. The log2 fold change in expression between blueberry and bilberry stage 7 flesh is shown. Mean counts represents the mean across all samples. Genes identified previously and used in further analysis are provided with gene names. The predicted function of the Arabidopsis gene is shown.

| **MSU Gene ID** | **Mapman Bin Annotation** | **Arabidopsis AtG** | **Annotation** | **Log2Fold** | **Mean Counts** | **Gene Name** | **Predicted function in Arabidopsis, if known** |
| --- | --- | --- | --- | --- | --- | --- | --- |
| vaccdscaff28-197.19 | MYB | AT4G38620.1 | MYB4 | 1.0 | 983 | MYBC2.1 | Repressor of phenylpropanoids ^1^ |
| vaccdscaff37-143.27 vaccdscaff38-163.31 vaccdscaff39-168.25  vaccdscaff6-261.32 | MYB | AT3G13540.1 | MYB5 | 8.0, 8.3, 7.8, 7.1 | 669, 2108, 3314, 913 | MYBPA1.1 | Controls outer seed coat differentiation, mucilage synthesis and trichome formation ^2,3^ |
| vaccdscaff9-307.24  vaccdscaff35-237.26  vaccdscaff4-330.20  vaccdscaff36-113.22 | MYB | AT4G09460.1 | MYB6 | 5.8, 5.5, 9.1, 5.0 | 192, 216, 216, 147 | MYBC2.2 | Repressor of flavonoids ^4^ |
| vaccdscaff58-1.25 | MYB | AT1G16490.1 | MYB58 | 2.2 | 85 |  | Activator of lignin biosynthesis ^5^ |
| vaccdscaff16-61.39  vaccdscaff7-363.26  vaccdscaff18-336.27 | MYB | AT3G47600.1 | MYB94 | 2.0, 3.5, 1.6 | 725, 274, 585 | MYB4 | Activator of cuticular wax biosynthesis ^6^ |
| vaccdscaff13-105.27 | MYB | AT1G66370.1 | MYB113 | 8.1 | 1543 | MYBA2 | Activator of anthocyanin biosynthesis ^7^ |
| vaccdscaff13-106.35 vaccdscaff1486-0.3 | MYB | AT1G66370.1 | MYB113 | 3.8, 4.3 | 1818, 4963 | MYBA1 | Activator of anthocyanin biosynthesis ^7^ |
| vaccdscaff1069-0.8 | MYB | AT1G01380.1 | ETC1 | 2.5 | 122 | MYBR3.2 | Repressor of trichome formation ^8^ |
| vaccdscaff190-0.40 | MED2/29/32 | AT1G11760.1 | MEDIATOR 2 | 3.6 | 60 |  | Regulates phenylpropanoid biosynthesis ^9^ |
| vaccdscaff11-136.2 | DOF | AT2G34140.1 | CDF4 | 4.0 | 32 |  | Cell differentiation ^10^ |
| vaccdscaff31-277.12 vaccdscaff18-68.36 | HD-ZIP I/II | AT4G40060.1 | HB16 | 4.5, 2.1 | 78, 57 |  |  |
| vaccdscaff43-73.22 | REM | AT1G49480.3 | RTV1 | 5.8 | 78 |  | Floral regulator ^11^ |
| vaccdscaff49-0.0  vaccdscaff49-3.6 | RAV/NGATHA | AT2G46870.1 | NGATHA1 | 2.9, 3.0 | 26, 33 |  | Induces ABA biosynthesis during drought stress ^12^ |
| vaccdscaff44-18.25 | NLP | AT4G24020.1 | NLP7 | 5.5 | 249 |  | Modulates nitrate sensing and metabolism ^13^ |
| vaccdscaff27-275.4  vaccdscaff17-311.1 | GRAS | AT1G07530.1 | SCL14 | 1.9, 3.2 | 198, 217 |  | Activates stress-responsive genes ^14^ |
| vaccdscaff32-313.21 | MADS/AGL | AT2G03710.2 | SEP4 | 1.9 | 507 | MADS1 | Involved in the development of sepals, petals, stamens and carpels ^15^ |
| vaccdscaff16-355.5 | C2H2 | AT1G27730.1 | STZ | 10.1 | 94 |  | Transcriptional repressor that is induced by several stress responses to inhibit plant growth ^16^ |
| vaccdscaff13-50.35  vaccdscaff30-305.45 | NAC | AT2G33480.1 | NAC041 | 4.6, 5.2 | 31, 45 | NAC1 | Involved in regulating mannan biosynthesis ^17^ |
| vaccdscaff24-191.23 vaccdscaff15-200.28 vaccdscaff19-273.26 vaccdscaff11-207.26 | WRKY | AT1G62300.1 | WRKY6 | 2.0, 1.7, 1.9, 2.7 | 434, 267, 294, 359 | WRKY3 | Involved in senescence, pathogen defense, low phosphate stress and ABA signalling ^18-20^ |
| vaccdscaff22-298.44 vaccdscaff24-64.33  vaccdscaff19-368.29 vaccdscaff43-79.16 | WRKY | AT2G38470.1 | WRKY33 | 3.7, 1.3, 3.0, 2.4 | 150, 387, 798, 833 | WRKY6 | Involved in fungal resistance ^21^ |
| vaccdscaff36-33.17  vaccdscaff9-377.26  vaccdscaff35-292.32  vaccdscaff4- 398.15 | WRKY | AT1G80840.1 | WRKY40 | 2.7, 3.2, 2.7, 4.5 | 1277, 1064, 1137, 2178 | WRKY5 | Pathogen and ABA induced transcriptional repressor which regulate plant defense to microbial pathogens and represses ABA responses ^22-24^ |
| vaccdscaff21-44.36 | WRKY | AT4G11070.1 | WRKY41 | 7.9 | 109 | WRKY2 | Transcriptional repressor of anthocyanins and activator of *AtABI3*, which regulates seed maturation ^25,26^ |
| vaccdscaff22-182.19 | JUMONJI | AT1G30810.3 | JMJ15 | 1.2 | 267 |  | A histone demethylase that regulates flowering time and high salt and temperature tolerance ^27-29^ |
| vaccdscaff27-261.27 vaccdscaff17-294.20 vaccdscaff34-85.27 | TAZ | AT5G63160.1 | BT1 | 1.3, 1.8, 1.2 | 278, 814, 968 |  | Transcriptional repressor of nitrate uptake genes and regulates plant development ^30,31^ |
| vaccdscaff11-350.30 vaccdscaff15-339.25 vaccdscaff24-51.34 | bHLH | AT2G43060.1 | IBH1 | 4.2, 1.7, 2.3 | 294, 611, 1569 |  | Transcriptional repressor of cell elongation ^32^ |
| vaccdscaff21-15.39  vaccdscaff33-304.30 vaccdscaff26-12.21  vaccdscaff29-11.26 | TIFY | AT1G19180.3 | JAZ1 | 2.3, 2.9, 2.2, 2.5 | 1620, 1700, 1426, 5017 |  | Repressors anthocyanin biosynthesis in the absence of jasmonate ^33^ |
| vaccdscaff16-197.18 | LUG | AT4G32551.1 | RON2 | 4.3 | 182 |  | Regulates flower and leaf development ^34,35^ |

**Supplementary Table S6.** Composition of tissue culture media used for blueberry transformation

|  | Micro-propagation | Co-cultivation | Selection | Shoot Proliferation | Rooting |
| --- | --- | --- | --- | --- | --- |
| WPM macronutrients^36^ | ✓ | ✓ | - | - | ✓ |
| Modified WPM macro /micro/Fe/vitamins^37^ | - | - | ✓ | ✓ | - |
| MS micronutrients^38^ | ✓ | ✓ | - | - | ✓ |
| Cupric sulfate | 5 µM | 5 µM | 5 µM | 5 µM | ✓ |
| MS iron^38^ | ✓ | ✓ | - | - | ✓ |
| B5 vitamins^39^ | ✓ | ✓ | - | - | ✓ |
| Zeatin | 4 mg L^-1^ | - | - | 4 mg L^-1^ | 0.3 mg L^-1^ |
| 1-naphthaleneacetic acid (NAA) | - | 0.5 mg L^-1^ | 0.5 mg L^-1^ | - | - |
| Thidiazuron (TDZ) | - | 1 mg L^-1^ | 1 mg L^-1^ | - | - |
| Coconut milk | - | - | - | - | 2% |
| Acetosyringone | - | 200 µM | - | - | - |
| Kanamycin sulfate | - | - | 30 mg L^-1^ | 30 mg L^-1^ | - |
| Cefotaxime | - | - | 250 mg L^-1^ | 250 mg L^-1^ | 250 mg L^-1^ |
| Sucrose | 20 g L^-1^ | 30 g L^-1^ | 30 g L^-1^ | 30 g L^-1^ | 20 g L^-1^ |
| Bacterial agar | 7.5 L^-1^ | 7.5 L^-1^ | 7.5 L^-1^ | 7.5 L^-1^ | - |
| Gelrite | - | - | - | - | 4 g L^-1^ |

1 Wang, X.-C. *et al.* Arabidopsis MYB4 plays dual roles in flavonoid biosynthesis. *The Plant Journal* **101**, 637-652, doi:<https://doi.org/10.1111/tpj.14570> (2020).

2 Gonzalez, A., Mendenhall, J., Huo, Y. & Lloyd, A. TTG1 complex MYBs, MYB5 and TT2, control outer seed coat differentiation. *Developmental Biology* **325**, 412-421, doi:<https://doi.org/10.1016/j.ydbio.2008.10.005> (2009).

3 Li, S. F. *et al.* The Arabidopsis MYB5 Transcription Factor Regulates Mucilage Synthesis, Seed Coat Development, and Trichome Morphogenesis  *The Plant Cell* **21**, 72-89, doi:10.1105/tpc.108.063503 (2009).

4 Lotkowska, M. E. *et al.* The Arabidopsis Transcription Factor MYB112 Promotes Anthocyanin Formation during Salinity and under High Light Stress. *Plant physiology* **169**, 1862-1880, doi:10.1104/pp.15.00605 (2015).

5 Zhou, J., Lee, C., Zhong, R. & Ye, Z.-H. MYB58 and MYB63 Are Transcriptional Activators of the Lignin Biosynthetic Pathway during Secondary Cell Wall Formation in Arabidopsis    *The Plant Cell* **21**, 248-266, doi:10.1105/tpc.108.063321 (2009).

6 Lee, S. B. & Suh, M. C. Cuticular Wax Biosynthesis is Up-Regulated by the MYB94 Transcription Factor in Arabidopsis. *Plant and Cell Physiology* **56**, 48-60, doi:10.1093/pcp/pcu142 (2014).

7 Gonzalez, A., Zhao, M., Leavitt, J. M. & Lloyd, A. M. Regulation of the anthocyanin biosynthetic pathway by the TTG1/bHLH/Myb transcriptional complex in Arabidopsis seedlings. *The Plant Journal* **53**, 814-827, doi:<https://doi.org/10.1111/j.1365-313X.2007.03373.x> (2008).

8 Kirik, V., Simon, M., Huelskamp, M. & Schiefelbein, J. The ENHANCER OF TRY AND CPC1 gene acts redundantly with TRIPTYCHON and CAPRICE in trichome and root hair cell patterning in Arabidopsis. *Developmental Biology* **268**, 506-513, doi:<https://doi.org/10.1016/j.ydbio.2003.12.037> (2004).

9 Dolan, W. L., Dilkes, B. P., Stout, J. M., Bonawitz, N. D. & Chapple, C. Mediator Complex Subunits MED2, MED5, MED16, and MED23 Genetically Interact in the Regulation of Phenylpropanoid Biosynthesis. *The Plant Cell* **29**, 3269-3285, doi:10.1105/tpc.17.00282 (2017).

10 Pi, L. *et al.* Organizer-Derived WOX5 Signal Maintains Root Columella Stem Cells through Chromatin-Mediated Repression of CDF4 Expression. *Developmental Cell* **33**, 576-588, doi:<https://doi.org/10.1016/j.devcel.2015.04.024> (2015).

11 Heo, J. B., Sung, S. & Assmann, S. M. Ca2+-dependent GTPase, Extra-large G Protein 2 (XLG2), Promotes Activation of DNA-binding Protein Related to Vernalization 1 (RTV1), Leading to Activation of Floral Integrator Genes and Early Flowering in Arabidopsis*. *Journal of Biological Chemistry* **287**, 8242-8253, doi:<https://doi.org/10.1074/jbc.M111.317412> (2012).

12 Sato, H. *et al.* *Arabidopsis thaliana* NGATHA1 transcription factor induces ABA biosynthesis by activating *NCED3* gene during dehydration stress. *Proceedings of the National Academy of Sciences*, 201811491, doi:10.1073/pnas.1811491115 (2018).

13 Castaings, L. *et al.* The nodule inception-like protein 7 modulates nitrate sensing and metabolism in Arabidopsis. *The Plant Journal* **57**, 426-435, doi:<https://doi.org/10.1111/j.1365-313X.2008.03695.x> (2009).

14 Fode, B., Siemsen, T., Thurow, C., Weigel, R. & Gatz, C. The Arabidopsis GRAS Protein SCL14 Interacts with Class II TGA Transcription Factors and Is Essential for the Activation of Stress-Inducible Promoters. *The Plant Cell* **20**, 3122-3135, doi:10.1105/tpc.108.058974 (2008).

15 Ditta, G., Pinyopich, A., Robles, P., Pelaz, S. & Yanofsky, M. F. The SEP4 Gene of Arabidopsis thaliana Functions in Floral Organ and Meristem Identity. *Current Biology* **14**, 1935-1940, doi:<https://doi.org/10.1016/j.cub.2004.10.028> (2004).

16 Sakamoto, H. *et al.* Arabidopsis Cys2/His2-Type Zinc-Finger Proteins Function as Transcription Repressors under Drought, Cold, and High-Salinity Stress Conditions. *Plant Physiology* **136**, 2734-2746, doi:10.1104/pp.104.046599 (2004).

17 Kim, W.-C. *et al.* Transcription factors that directly regulate the expression of CSLA9 encoding mannan synthase in Arabidopsis thaliana. *Plant Molecular Biology* **84**, 577-587, doi:10.1007/s11103-013-0154-9 (2014).

18 Huang, Y., Feng, C.-Z., Ye, Q., Wu, W.-H. & Chen, Y.-F. Arabidopsis WRKY6 Transcription Factor Acts as a Positive Regulator of Abscisic Acid Signaling during Seed Germination and Early Seedling Development. *PLOS Genetics* **12**, e1005833, doi:10.1371/journal.pgen.1005833 (2016).

19 Chen, Y.-F. *et al.* The WRKY6 transcription factor modulates PHOSPHATE1 expression in response to low Pi stress in Arabidopsis. *The Plant cell* **21**, 3554-3566, doi:10.1105/tpc.108.064980 (2009).

20 Robatzek, S. & Somssich, I. E. Targets of AtWRKY6 regulation during plant senescence and pathogen defense. *Genes & development* **16**, 1139-1149 (2002).

21 Zheng, Z., Qamar, S. A., Chen, Z. & Mengiste, T. Arabidopsis WRKY33 transcription factor is required for resistance to necrotrophic fungal pathogens. *The Plant Journal* **48**, 592-605, doi:<https://doi.org/10.1111/j.1365-313X.2006.02901.x> (2006).

22 Chen, H. *et al.* Roles of arabidopsis WRKY18, WRKY40 and WRKY60 transcription factors in plant responses to abscisic acid and abiotic stress. *BMC Plant Biology* **10**, 281, doi:10.1186/1471-2229-10-281 (2010).

23 Liu, Z.-Q. *et al.* Cooperation of three WRKY-domain transcription factors WRKY18, WRKY40, and WRKY60 in repressing two ABA-responsive genes ABI4 and ABI5 in Arabidopsis. *Journal of Experimental Botany* **63**, 6371-6392, doi:10.1093/jxb/ers293 (2012).

24 Xu, X., Chen, C., Fan, B. & Chen, Z. Physical and Functional Interactions between Pathogen-Induced Arabidopsis WRKY18, WRKY40, and WRKY60 Transcription Factors. *The Plant Cell* **18**, 1310-1326, doi:10.1105/tpc.105.037523 (2006).

25 Duan, S. *et al.* Functional characterization of a heterologously expressed Brassica napus WRKY41-1 transcription factor in regulating anthocyanin biosynthesis in Arabidopsis thaliana. *Plant Science* **268**, 47-53, doi:<https://doi.org/10.1016/j.plantsci.2017.12.010> (2018).

26 Ding, Z. J. *et al.* WRKY41 controls Arabidopsis seed dormancy via direct regulation of ABI3 transcript levels not downstream of ABA. *The Plant Journal* **79**, 810-823, doi:<https://doi.org/10.1111/tpj.12597> (2014).

27 Cui, X., Zheng, Y., Lu, Y., Issakidis-Bourguet, E. & Zhou, D.-X. Metabolic control of histone demethylase activity involved in plant response to high temperature. *Plant Physiology* **185**, 1813-1828, doi:10.1093/plphys/kiab020 (2021).

28 Yang, H. *et al.* Overexpression of a histone H3K4 demethylase, JMJ15, accelerates flowering time in Arabidopsis. *Plant Cell Reports* **31**, 1297-1308, doi:10.1007/s00299-012-1249-5 (2012).

29 Shen, Y. *et al.* Over-expression of histone H3K4 demethylase gene JMJ15 enhances salt tolerance in Arabidopsis. *Frontiers in Plant Science* **5**, doi:10.3389/fpls.2014.00290 (2014).

30 Araus, V. *et al.* Members of BTB Gene Family of Scaffold Proteins Suppress Nitrate Uptake and Nitrogen Use Efficiency. *Plant Physiology* **171**, 1523-1532, doi:10.1104/pp.15.01731 (2016).

31 Robert, H. S., Quint, A., Brand, D., Vivian-Smith, A. & Offringa, R. BTB and TAZ domain scaffold proteins perform a crucial function in Arabidopsis development. *The Plant Journal* **58**, 109-121, doi:<https://doi.org/10.1111/j.1365-313X.2008.03764.x> (2009).

32 Zhang, L.-Y. *et al.* Antagonistic HLH/bHLH Transcription Factors Mediate Brassinosteroid Regulation of Cell Elongation and Plant Development in Rice and Arabidopsis      *The Plant Cell* **21**, 3767-3780, doi:10.1105/tpc.109.070441 (2009).

33 Qi, T. *et al.* The Jasmonate-ZIM-Domain Proteins Interact with the WD-Repeat/bHLH/MYB Complexes to Regulate Jasmonate-Mediated Anthocyanin Accumulation and Trichome Initiation in Arabidopsis thaliana    *The Plant Cell* **23**, 1795-1814, doi:10.1105/tpc.111.083261 (2011).

34 Cnops, G. *et al.* The rotunda2 mutants identify a role for the LEUNIG gene in vegetative leaf morphogenesis. *J Exp Bot* **55**, 1529-1539, doi:10.1093/jxb/erh165 (2004).

35 Liu, Z. & Meyerowitz, E. M. LEUNIG regulates AGAMOUS expression in Arabidopsis flowers. *Development* **121**, 975-991 (1995).

36 Lloyd, G. & McCown, B. H. Commercially-feasible micropropagation of mountain laurel, Kalmia latifolia, by use of shoot-tip culture. *Combined Proceedings, International Plant Propagators' Society* **30**, 421-427 (1980).

37 Rowland, L. J. & Ogden, E. L. Efficient shoot regeneration from leaf sections of highbush blueberry suitable for use in *Agrobacterium*-mediated transformations. *ActaHortic.*, 193-198, doi:10.17660/ActaHortic.1993.336.24 (1993).

38 Murashige, T. & Skoog, F. A revised medium for rapid growth and bio assays with tobacco tissue cultures. *Physiologia Plantarum* **15**, 473-497, doi:<https://doi.org/10.1111/j.1399-3054.1962.tb08052.x> (1962).

39 Gamborg, O. L., Miller, R. A. & Ojima, K. Nutrient requirements of suspension cultures of soybean root cells. *Exp Cell Res* **50**, 151-158, doi:10.1016/0014-4827(68)90403-5 (1968).
